# Seasons and shape: inflorescences from autumn to summer

**DOI:** 10.1093/jxb/eraf261

**Published:** 2025-07-01

**Authors:** Pablo González-Suárez, Thomas Lock, Steven Penfield, Jo Hepworth

**Affiliations:** Department of Developmental Genetics, Centre for Plant Molecular Biology (ZMBP), Eberhard Karls University, Tuebingen, Germany; Department of Crop Genetics, John Innes Centre, Norwich Research Park, Norwich, UK; Department of Crop Genetics, John Innes Centre, Norwich Research Park, Norwich, UK; Department of Biosciences, Durham University, Durham, UK; University College Dublin, Ireland

**Keywords:** branching, flowering, inflorescence, meristem, plant reproductive development, temperature

## Abstract

Flowering plants organize their reproductive organs within specialized structures named inflorescences. Plasticity in the architecture of these inflorescences allows adaptation to the environment during flowering, ultimately determining reproductive output and yield. Inflorescence development relies on meristems, hubs of pluripotent cells that direct organogenesis. In recent years, laboratory studies have uncovered the response of meristems and their resulting inflorescences to environmental cues such as temperature, which is subject to both unpredictable and seasonal fluctuations. In this review, we explore the mechanisms through which temperature regulates inflorescence development in both model and crop species, principally from the *Brassicaceae* family. We follow the trajectory of the apical meristem through the seasons, from acquisition of reproductive identity to arrest and including branch outgrowth, highlighting the current understanding of the mechanisms through which temperature influences development. While the role of temperature in regulating the floral transition has been well established, we emphasize significant gaps in our understanding of how subsequent developmental transitions are controlled. Furthermore, we find that many key gene networks underpinning inflorescence development were initially characterized in Arabidopsis under controlled laboratory conditions. However, recent studies in other *Brassicaceae* species and crops have revealed that our understanding of gene function in the field context remains limited, posing a challenge for breeding efforts aimed at climate resilience.

## Teaser

We review temperature regulation of reproductive development in seasonal contexts, focusing on the molecular factors controlling change at meristems from vegetative growth, via production of the inflorescence and branches, to the end of flowering.

## Introduction

Angiosperms are defined by their flowers, and these highly varied and complex structures are largely credited with the rapid expansion of angiosperm species. However, the plant architectures that support these flowers are also highly varied in their forms. Typically, flowers are arranged in specialized structures named inflorescences, whose shape and architecture vary greatly between and within species. Inflorescences can make up the majority of the plant body after flowering, especially in monocarpic species such as *Arabidopsis thaliana*, despite representing only the reproductive phase of a plant’s life. As such, their size, rate of progression through developmental transitions, and resultant architecture have important effects on fitness in the wild and yield on the farm.

Most plant development is environmentally responsive, but developmental phase transitions, such as seed germination or the floral transition, are under particularly tight environmental control. In temperate regions, strong seasonal change provides both drivers and cues for changes in development. This has made initiation of the floral transition, particularly in Arabidopsis, a paradigm for studying the genetic underpinning of temperature and daylength responses. However, for a long time it has been recognized that the creation of inflorescences involves a sequence of distinct developmental transitions, from cessation of vegetative leaf production to final floral meristem (FM) formation, whereby different structures are formed over time in response to specific environmental and endogenous cues (Suh *et al*., 2003; Prusinkiewicz *et al*., 2007). Thus, temporal and spatial variation in the rate at which progression through these transitions occurs is known to have critical effects on the shape of the inflorescence (Azpeitia *et al*., 2021). Most of these effects rely on developmental decisions taken at the inflorescence meristems (IMs), niches of undifferentiated cells that govern the production of flowers and, ultimately, the final architecture of the inflorescence (Benlloch *et al*., 2007).

In this review, we will discuss what is known about temperature response throughout the development of the inflorescence, picking up as the meristem acquires inflorescence identity and following it through flowering branch production to cessation of flower production, although we will not discuss flower development itself which is covered in detail elsewhere (Heisler *et al*., 2022). We will use an Arabidopsis framework to discuss the conserved transitions between meristem and organ identities, but draw on examples from different dicot species, primarily from the *Brassicaceae* family.

## Reproductive structures and identities

Inflorescences are produced by the shoot apical meristem (SAM). Vegetative SAMs are indeterminate and iteratively produce phytomers, structural units of the plant containing leaves and axillary meristems (AMs), usually separated by internodes. The floral transition results in an IM that initiates determinate FMs, each one of which holds the potential to become a flower and, later, a seed-bearing fruit. The rate of progression from a vegetative meristem to a determinate FM therefore influences the number of branch nodes available to the plant. However, as discussed already, this is a multistage transition with different environmental and developmental requirements at each stage (King, 2015). Moreover, AMs can acquire inflorescence identity as well, producing secondary and higher order inflorescence branches. When and how these multiple reproductive meristems arrest their growth further controls the shape of the inflorescence. Additionally, for polycarpic perennials which flower multiple times during their lifetime, this shape is not final, and growth arrest is replaced with developmental reprogramming to resume vegetative growth until the next reproductive season. Thus, there are many processes that influence the architecture of inflorescences over the seasons.

**Fig. 1.**
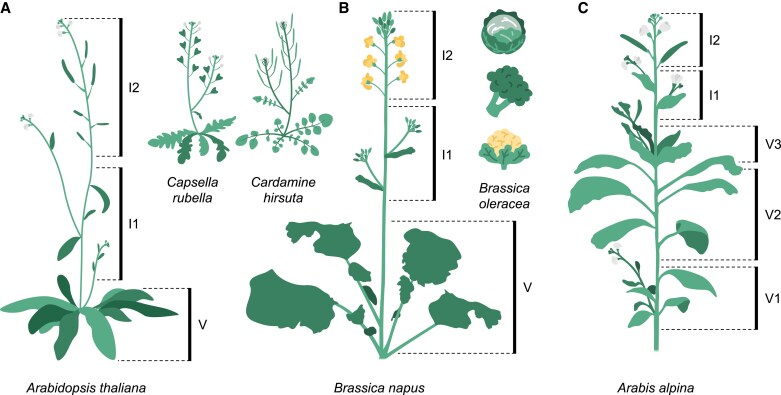
Variety of architectures in the flowering *Brassicaceae* family. (A, B) Typical inflorescence zonation in annuals, weeds (A), and crops (B). Most vegetative organs formed prior to flowering are in the V zone. In the inflorescence, the basal I1 zone holds axillary buds and branches, and the apical I2 zone sustains solitary fruits, flowers, and floral buds. (C) Typical inflorescence zonation in perennials. The vegetative region can be divided into a V1 with axillary inflorescences, a V2 with dormant axillary buds, and a V3 with subapical vegetative branches. As in annuals, I1 and I2 are found in the inflorescence.

In dicots from temperate regions, the raceme is a common inflorescence type (Benlloch *et al*., 2007; Prusinkiewicz *et al*., 2007). In the *Brassicaceae* family, this raceme is composed of a main axis stemming from the vegetative zone (V) which bears either flowers or lateral axes (i.e. axillary inflorescences), which in turn reiterate this morphology (Prusinkiewicz *et al*., 2007). Arabidopsis and other annual species from the *Brassica* genus form one of the simplest racemes, with two zones characterized by the development of different organs (Fig. 1A). The inflorescence zone produced after the initial reproductive transition (I1) contains leaf phytomers with AMs which can grow out into inflorescence branches. The I2 zone holds the primary inflorescence, where solitary flowers are initiated on the periphery of the meristem and leaf production is suppressed (Ratcliffe *et al*., 1998; Lazaro *et al*., 2018). Inflorescence branches from the I1 follow a similar developmental trajectory to the main stem, whereby they themselves can produce subordinate AMs (also I1) before starting flower production (I2). However, these higher order AMs are more likely to be quiescent (Azpeitia *et al*., 2021).

The zonation of the inflorescence has important implications in agriculture. This is best illustrated by the diverse crop *B. oleracea*, whose subspecies can take on a wide array of different vegetable forms depending on which inflorescence architecture is selected for (Fig. 1B). For example, cabbages (*B. oleracea* ssp. *capitata*) are a head of packed vegetative leaves enclosing the meristem, which are ultimately derived from the V region (Alemán-Báez *et al*., 2022). On the other hand, cauliflowers (*B. oleracea* ssp. *botrytis*) are a dense cluster of arrested reproductive meristems whose floral primordia do not develop into flowers, suggesting that the distinctive curd is analogous to the I1 zone (Sadik, 1962). Finally, broccoli (*B. oleracea* ssp. *italica*), which is made up of packed florets, arises from the I2 zone (Gray, 1982).

In addition to the typical V/I1/I2 zonation of *A. thaliana*, longer-lived relatives such as the polycarpic *Arabis alpina* contain additional V zones bearing secondary structures with different identities, all of which are initiated prior to the appearance of the apical inflorescence (R. Wang *et al*., 2009; Lazaro *et al*., 2018; Vayssières *et al*., 2020) (Fig. 1C). The V1, typically composed of the earliest developed meristems and often formed before winter, gives rise to axillary flowering branches. In contrast, the V3 zone, usually initiated during the cold period, holds subapical vegetative branches. Plants exposed to a sufficiently long period of cold form a V2 zone in between, containing a series of buds that are typically kept dormant during flowering (Vayssières *et al*., 2020). The difference in developmental trajectories among these different structures stems from the asynchronous development of their source AMs, with those from I1, I2, and V1 being the only ones having experienced enough cold, and being of the right age, to acquire inflorescence identity through the process of vernalization (Lazaro *et al*., 2018; Hyun *et al*., 2019; Vayssières *et al*., 2020). In turn, AMs of zones V2 and V3 are critical to perenniality, as they do not senesce after reproduction and allow for new vegetative growth the following season (Vayssières *et al*., 2020).

## The making of inflorescence meristems

**Fig. 2.**
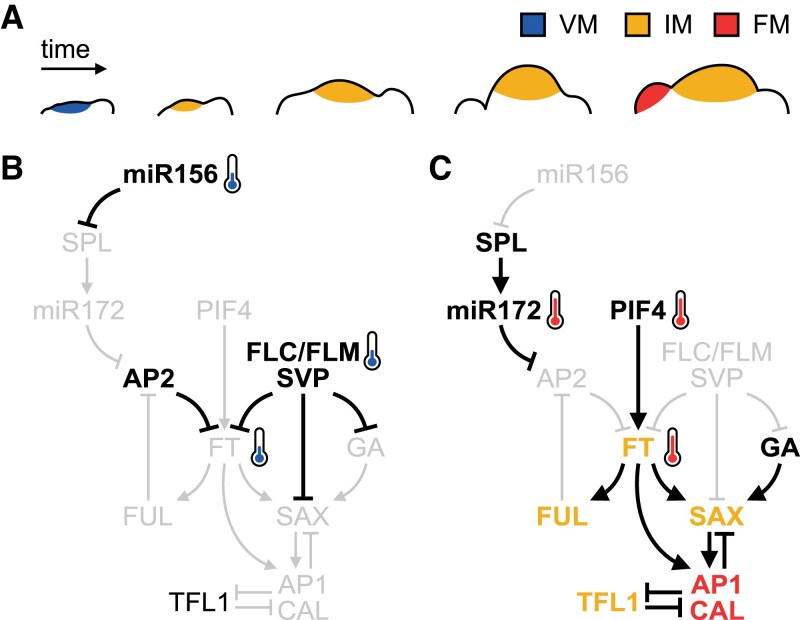
Several temperature inputs converge at the floral integrators. (A) Diagram depicting morphological changes during the conversion of a vegetative SAM (VM) to an inflorescence meristem (IM), and the subsequent initiation of floral meristems (FMs). (B, C) Network summarizing the major molecular interactions discussed throughout the text which occur under cold (B) and warm (C) temperatures during this transition. Arrows and blunt arrows indicate activation and repression, respectively. Thermometers highlight temperature inputs into the system. Genes in grey indicate components that are inactive at the respective temperatures, and colours indicate factors relevant to a specific meristem identity.

The molecular bases of inflorescence formation have been studied in detail in model plants, leading to the identification of a complex regulatory network which converges on to a subset of conserved regulators and markers of floral transitions (Fig. 2A). In Arabidopsis these are principally two modules of homologous MADS-box transcription factors, plus the unique transcription factor LEAFY (LFY). Expression of the ‘inflorescence integrators’ SUPPRESSOR OF OVEREXPRESSION OF CONSTANS1, AGAMOUS-LIKE24, and XAANTAL2 (SOC1, AGL24, and XAL2; the ‘SAX’ MADS-box module) leads to up-regulation of the second module, formed of FM markers APETALA1 and its homologue CAULIFLOWER (AP1/CAL), which negatively regulates SAX expression in a feedback loop (Liu *et al*., 2007; Immink *et al*., 2012; Pajoro *et al*., 2014; Azpeitia *et al*., 2021) (Fig. 2B, C). Expression of these regulators at the meristem or in the subtending primordia are hallmarks of inflorescence identity. Thus, their interactions define the spatial and temporal developmental progress of the IM and its floral primordia through to determinacy and cessation.

Many elements of the floral transition network upstream of these factors are temperature sensitive, but two main pathways primarily relay temperature information: the vernalization pathway and the ambient temperature or thermosensory pathway (Srikanth and Schmid, 2011; Song *et al*., 2015). The vernalization pathway acts to prevent plants that germinate in the warmer months from opening flowers until the following spring, being most active in Arabidopsis accessions with a winter annual (sometimes ‘biennial’) life cycle. This is believed to be the ancestral life history, with most rapid cycling accessions (including the commonly used Col-0 and L*er-*1) having low, though functional, expression levels of the main vernalization regulator *FLOWERING LOCUS C* (*FLC*) (Johanson *et al*., 2000; Gazzani *et al*., 2003; Whittaker and Dean, 2017). As a result, much knowledge of the floral transition network has been established in these low-*FLC* backgrounds, in warm and long-lighting conditions and so in a ‘spring’-like genetic and environmental context.

### Spring and summer pathways

Two key integrators of environmental signals throughout the floral transition are the ‘florigen’ phosphatidylethanolamine-binding proteins (PEBPs) FLOWERING LOCUS T (FT) and TWIN SISTER OF FT (TSF). For FT in particular, the key environmental cue for its expression is photoperiod, the mechanisms of which have been extensively and well reviewed elsewhere (Song *et al*., 2015). Under promotive conditions, *FT* and *TSF* are expressed in the phloem companion cells in leaves (Kobayashi *et al*., 1999; Wigge *et al*., 2005; Yamaguchi *et al*., 2005; Chen *et al*., 2018). The resulting proteins are loaded into the phloem and moved towards the apex (Corbesier *et al*., 2007; Jaeger and Wigge, 2007; Mathieu *et al*., 2007). At the apex, they form a regulatory complex with the bZIP transcription factor FD which transcriptionally activates genes that specify the identity of the developing IM as well as of the floral primordia that it will form in its periphery (Abe *et al*., 2005; Wigge *et al*., 2005; Yoo *et al*., 2005). Within this complex molecular architecture, the bulk of the temperature signal integration into the flowering programme involves regulation of *FT* at various levels.

The thermosensory pathway dominates the transcriptional control of *FT* by temperature as characterized within a range of ∼10–27 °C, which has been reviewed in detail previously (Susila *et al*., 2018). The MADS-box transcription factor SHORT VEGETATIVE PHASE (SVP) has a key role in this process (Blázquez *et al*., 2003; Lee *et al*., 2013). In colder temperatures, SVP represses *FT* transcription both directly (Lee *et al*., 2007; Li *et al*., 2008) and indirectly, for example via activation of the *TEMPRANILLO* (*TEM*) genes (Castillejo and Pelaz, 2008; Marín-González *et al*., 2015). When acting directly, SVP interacts with itself and other MADS-box transcription factors including the MADS AFFECTING FLOWERING (MAF1–5) family to form homo- and heterotetramers that bind to the promoter of *FT* and *TSF* to repress their expression (Lee *et al*., 2007, 2013; Gu *et al*., 2013; Posé *et al*., 2013). Expression of *SVP* is only moderately affected by temperature (Lee *et al*., 2007; Posé *et al*., 2013), but *MAF1*, also commonly known as *FLOWERING LOCUS M* (*FLM*), is strongly up-regulated in the cold (Lee *et al*., 2013; Sureshkumar *et al*., 2016). Additionally, cold promotes the production of a specific spliceform, FLM-β, which seems to favour the binding of SVP to its targets (Balasubramanian *et al*., 2006; Lee *et al*., 2013; Posé *et al*., 2013; Sureshkumar *et al*., 2016). Indeed, complexation with FLM-β maintains SVP in the nucleus, while down-regulation of *FLM-β* in the warmth triggers its translocation to the cytosol, where it can be marked for proteasomal degradation (Jin *et al*., 2022). When present, a further MADS-box transcription factor controls the repression of *FT*; namely FLC. Like its MAF relatives, FLC dimerizes with SVP (Li *et al*., 2008) and binds to CArG box motifs in the genomic regions of *FT* and *TSF* to inhibit their transcription (Yamaguchi *et al*., 2005; Searle *et al*., 2006; Lee *et al*., 2007). Besides its transcriptional control, FT is post-translationally repressed in the cold through sequestration in intracellular membranes, where it interacts with phospholipids (Liu *et al*., 2020; Susila *et al*., 2021) or proteins (Susila *et al*., 2024). This compromises the long-range mobility of FT, further contributing to the delay of the floral transition at lower temperatures.

**Fig. 3.**
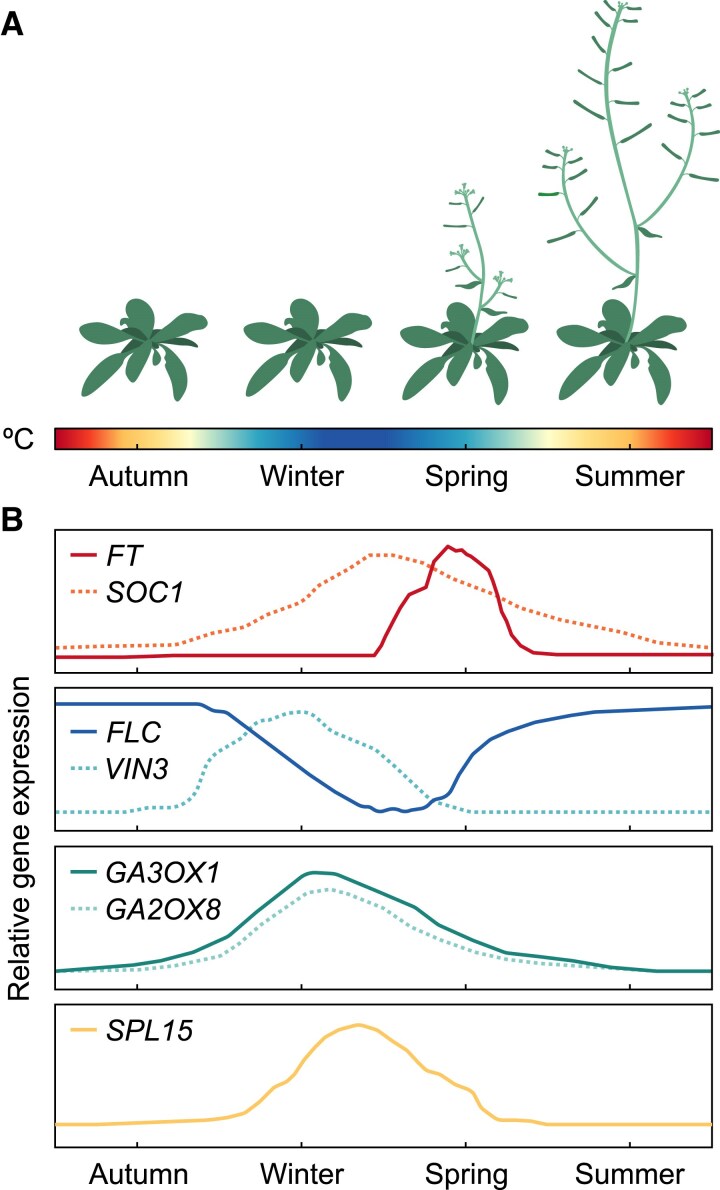
Seasonal fluctuations in the transcription of key floral integrators and their upstream regulators. (A) Cartoon depicting development of a generic perennial from the *Brassicaceae* family exposed to seasonal temperature fluctuations. Bolting is assumed to occur in spring, and flowering to cease in summer. (B) Line graphs illustrating seasonal changes in expression of key genes involved in inflorescence development. Data taken from Satake *et al*. (2013) and Nagano *et al*. (2019).

In addition to the repressors that govern the control of *FT* in the cold, several activators promote its transcription upon warming. Such is the case of the basic helix–loop–helix (bHLH) transcription factors PHYTOCHROME-INTERACTING FACTOR 4 (PIF4) and PIF5 (Kumar *et al*., 2012; Thines *et al*., 2014). Different mechanisms have been proposed to explain *PIF4* induction in the warmth, including direct activation by TEOSINTE BRANCHED1/CYCLOIDEA/PCF TRANSCRIPTION FACTOR 5 (TCP5) (Han *et al*., 2019) and BRASSINAZOLE-RESISTANT 1 (BZR1), whose nuclear localization is temperature dependent (Ibañez *et al*., 2018). The evening complex of the circadian clock, composed of LUX ARRHYTHMO (LUX), EARLY FLOWERING 3 (ELF3), and ELF4, also controls *PIF4* expression directly (Nusinow *et al*., 2011; Ezer *et al*., 2017), with a specific prion domain of ELF3 acting as a temperature sensor (Jung *et al*., 2020). Lastly, histone deacetylation has a role in the regulation of *PIF4* and its targets (Tasset *et al*., 2018). The histone variant H2A.Z exhibits increased chromatin eviction at high ambient temperatures in Arabidopsis, increasing DNA accessibility and therefore expression of enclosed genes (Kumar and Wigge, 2010; Shen *et al*., 2019; van der Woude *et al*., 2019). This is mediated by temperature-dependent deacetylation of H2A.Z by POWERDRESS (PWR) and HISTONE DEACETYLASE9 (HDA9), ultimately leading to increased transcriptional competence of the *PIF4* locus at warm ambient temperatures. Consequently, cold repression and promotion in warm long photoperiods ensure that, in the field, FT is a signature of flowering in spring and summer (Fig. 3).

### A flower for all seasons: other temperature-sensitive pathways

Although the major temperature inputs to IM formation are usually conceptualized as coming through the vernalization and thermosensory pathways, other flowering pathways are temperature sensitive. One such example is the age pathway, which ensures that plants only flower after reaching maturity. Briefly, this involves the opposing activity of two miRNAs, miR156 and miR172. In juvenile plants, the highly abundant miR156 translationally inhibits *SQUAMOSA PROMOTER BINDING-LIKE* (*SPL*) transcription factors, which themselves can promote expression of miR172 (Wu and Poethig, 2006; Gandikota *et al*., 2007; J.-W. Wang *et al*., 2009; Wu *et al*., 2009) (Fig. 2B, C). As the plant ages into maturity, miR156 levels decline via histone-mediated repression linked to cell division, itself inherently sensitive to temperature-responsive changes in growth rates (Cheng *et al*., 2021). The decline in miR156 leads to derepression of *SPL* genes and subsequent induction of their targets including miR172, which in turn promote flowering by inhibiting the activity of reproductively repressive *APETALA2* (*AP2*)-like genes such as *TARGET OF EAT1* (*TOE1*) and *TOE2* (Aukerman and Sakai, 2003; Chen, 2004; Wu and Poethig, 2006; Wu *et al*., 2009). *SPL15* promotes flowering in the absence of both *FT* and *TSF* (Hyun *et al*., 2019), but SPL3 also directly up-regulates *FT* (Kim *et al*., 2012), such that the age pathway integrates within the floral network at multiple levels. Intriguingly, miR156 and miR172 exhibit opposing thermosensory responses: miR156 is up-regulated at cool ambient temperatures, probably through regulation at the RNA processing level (Kim *et al*., 2016), whilst miR172 is up-regulated at warm ambient temperatures (H. Lee *et al*., 2010), at least partly through the activity of the RNA-binding protein FLOWERING CONTROL LOCUS A (FCA) which promotes processing of primary-miR172 to mature miR172 (Jung *et al*., 2012). In Arabidopsis, variability in flowering responses to warm temperatures is dependent on regulation of the miR172 level, which is sensitive to mutations in *SVP* (H. Lee *et al*., 2010). In fact, both SVP and FLM bind to the promoter of *miR172a* (Cho *et al*., 2012; Tao *et al*., 2012; Posé *et al*., 2013), generating crosstalk between the thermosensory and age pathways in the regulation of reproductive development.

Another essential facet to IM development is the potent promotive activity of gibberellin (GA). GAs are a family of growth-inductive plant hormones with wide-ranging functions in plants which have long been established in promoting flowering (Rieu *et al*., 2008; Mutasa-Göttgens and Hedden, 2009). In brief, GAs regulate development by modulating the activity of the DELLA family of growth repressors. In Arabidopsis, there are five DELLAs, GA INSENSITIVE (GAI), REPRESSOR OF GAI (RGA), RGA-LIKE1 (RGL1), RGL2, and RGL3, all of which negatively regulate the activity of transcription factors including PIF4 and the SPLs (Peng *et al*., 1997; Lee *et al*., 2002; K.P. Lee *et al*., 2010; Tyler *et al*., 2004; de Lucas *et al*., 2008; Yu *et al*., 2012). In this pathway, bioactive GAs bind to the receptor GIBBERELLIN INSENSITIVE DWARF1 (GID1), leading to a conformational change which permits interaction with DELLA proteins, culminating in proteasomal degradation of the complex (Silverstone *et al*., 2001; Ueguchi-Tanaka *et al*., 2005; Griffiths *et al*., 2006; Willige *et al*., 2007; Murase *et al*., 2008; Shimada *et al*., 2008). Through degradation of DELLAs, which otherwise have inhibitory effects on development, GAs act as global regulators of many processes, for example floral transition and stem elongation (King *et al*., 2001; Cheng *et al*., 2004; Tyler *et al*., 2004; Rieu *et al*., 2008; Yu *et al*., 2012), and thus regulation of GA synthesis in response to environmental cues is critical for appropriate inflorescence development. Sensitivity to temperature is in part achieved through FLC and SVP, which can act independently and coordinately to repress GA biosynthesis genes (Andrés *et al*., 2014; Mateos *et al*., 2015). These include the GIBBERELLIN 3-OXIDASE (GA3OX) and GIBBERELLIN 20-OXIDASE (GA20OX) family, which function in the final stages of GA biosynthesis (Hedden, 2020). Additionally, TEM1 also exerts photoperiodic control over *GA3OX1* and *GA3OX2* by direct interaction with *cis-*regulatory elements (Osnato *et al*., 2012). That being said, the expression dynamics of GA metabolism genes in winter annuals under field conditions remain poorly characterized. In contrast, a study on the overwintering perennial *Arabidopsis halleri* revealed clear seasonal patterns of gene expression; for instance, *GA2OX8* and *GA3OX1* are strongly up-regulated during winter (Fig. 3B), coinciding with the repression of *FLC* (Komoto *et al*., 2024). In an annual Arabidopsis, it has been shown that GA only has minor contributions to flowering time under long days, but under short days it becomes the primary, essential, component (Wilson *et al*., 1992; Hisamatsu and King, 2008; Rieu *et al*., 2008). Taken together, these data suggest an important function for GAs in the early development of the inflorescence as floral initiation in overwintering plants often occurs during or before the winter (see ‘Floral buds are winter structures’). Furthermore, GAs positively regulate the floral integrators *SOC1* (Moon *et al*., 2003; Porri *et al*., 2012) and *LFY* (Blázquez *et al*., 1998; Eriksson *et al*., 2006), which may provide a mechanism for the initiation of flowering in short days in the absence of *FT* and *TSF*.

### Taking the slow road to flowering

For many monocarpic plants, life begins not in spring, but in summer or autumn, a life history that may allow plants to make full use of the year by growing through late autumn and winter. Gene variants promoting this life history show signatures of selection linking it to higher latitudes and altitudes, where growing seasons may be shorter (Lempe *et al*., 2005; Ågren *et al*., 2017). High *FLC* expression delays flowering in a quantitative manner in autumn, ensuring that winter annuals do not flower until they have experienced a prolonged period of cold (Lempe *et al*., 2005; Shindo *et al*., 2005; Hepworth *et al*., 2020). In Arabidopsis, this pathway is mainly active in accessions with functional alleles of *FRIGIDA* (*FRI*), a transcriptional activator that up-regulates expression of *FLC* early in development (Michaels and Amasino, 1999; Johanson *et al*., 2000). During autumn and winter, cold represses *FLC* expression through a network of temperature-sensitive upstream interactions (Antoniou-Kourounioti *et al*., 2018). These include various mechanisms such a sequestration of FRI into nuclear condensates, particularly during cold nights (Zhu *et al*., 2021), freezing-triggered up-regulation of the antisense long non-coding RNA *COOLAIR* (Zhao *et al*., 2021), and particularly the nucleation of repressive chromatin-remodelling complexes at the *FLC* locus triggered by the cold-induced PHD protein VERNALIZATION INSENSITIVE 3 (VIN3) (Sung and Amasino, 2004; De Lucia *et al*., 2008; Bond *et al*., 2009). The chromatin environment triggered during winter at the *FLC* locus maintains repression of *FLC* even when plants are returned to the warm, releasing repression of *FT*, *SOC1*, *SPL15*, and other floral integrators until the process is reset in the following generation. The regulation of *FLC* is highly complex, and is reviewed in more detail elsewhere (Whittaker and Dean, 2017), but it is an unusual case in that the range and response speed of the principal temperature inputs have been mapped in field-relevant temperatures (Antoniou-Kourounioti *et al*., 2018; Zhu *et al*., 2022). At least three temperature pathways control *VIN3* expression alone, including clock-mediated pathways (Antoniou-Kourounioti *et al*., 2018; Hepworth *et al*., 2018; Kyung *et al*., 2022), rapid heat-sensitive repression pathways that prevent accumulation of *VIN3* during warm autumn days (Sung and Amasino, 2004; Hepworth *et al*., 2018), and the very slow promotion of *VIN3* via increased concentration of NTL8 protein due to reduced cell division (Zhao *et al*., 2020). Thus, slower growth in the cold is directly linked to reproductive development in a manner analogous to cell division-dependent *miR156* repression in the warmth.

## Floral buds are winter structures

**Fig. 4.**
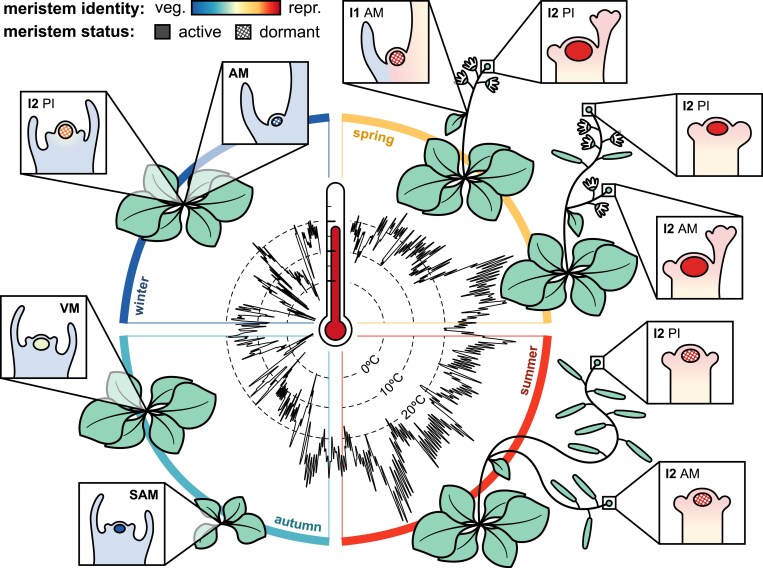
Changes in meristem activity and identity across the seasons. Circular diagram illustrating the multiple developmental transitions that occur in aerial meristems of Arabidopsis during the year. The temperature profile and timing of events are based on a field trial conducted in Norwich, UK (Lu *et al*., 2022). The apical vegetative meristem (VM) acquires reproductive identity during late autumn and early winter, after which it initiates floral primordia and later becomes dormant. In spring, flowering-promotive conditions trigger bolting, and the VM from the primary inflorescence (PI), now fully transitioned into an inflorescence meristem (IM), resumes reproductive development in the I2 zone of the inflorescence. Although delayed in their floral transition, axillary meristems (AMs) follow a similar developmental fate in the I1 zone. Finally, IMs undergo proliferative arrest in summer.

Although vernalization is often referred to as being a ‘winter’ pathway (usually studied under constant 5 °C in the lab), in both dicots and monocots vernalization pathways function in the mid to high teens (Wollenberg and Amasino, 2012; Duncan *et al*., 2015; Dixon *et al*., 2019). In Arabidopsis, cool nights below 14 °C are sufficient for transient transcriptional repression of *FLC*, with epigenetic repression pathways triggered quantitatively when daily highs fall below ∼15 °C (Antoniou-Kourounioti *et al*., 2018; Hepworth *et al*., 2018). As shown in Fig. 4, this autumnal vernalization permits the floral transition to occur before winter in field-grown Arabidopsis and related winter annual crops such as *B. napus*, despite the absence of *FT*, due to the release from FLC repression of floral integrators such as *SOC1* and *SPL5* (O’Neill *et al*., 2019; Matar *et al*., 2021; Lu *et al*., 2022). The perennial *A. alpina* will also initiate a set of IMs in the cold during autumn following extended vernalization thanks to silencing of the *FLC* orthologue *PERPETUAL FLOWERING 1* (*PEP1*) and up-regulation of orthologues of *SOC1*, *FRUITFUL* (*FUL*), and *SPL15* (R. Wang *et al*., 2009; Lazaro *et al*., 2018; Hyun *et al*., 2019). These IMs produce floral buds that remain dormant until spring, while insufficiently vernalized AMs produced later in the season remain vegetative until the flowering season in the following year, a state reinforced by the reactivation of *PEP1* in spring (R. Wang *et al*., 2009). In the apex, floral transition after vernalization is marked by up-regulation of *LFY*, *FUL*, and *AP1* (Lazaro *et al*., 2018). Preceding this, *SOC1* is up-regulated in both inflorescence and vegetative meristems (Lazaro *et al*., 2018; Matar *et al*., 2021). However, in perennial and winter annual *Brassicaceae*, bolting (stem extension) and anthesis do not occur until spring with the arrival of longer days and warmer temperatures. Therefore, there is a developmental window over the winter where the IM has initiated floral primordia but is maintained in a dormancy-like state, whereby elongation of reproductive tissues is inhibited (Fig. 4). This observation suggests that the winter annual life history in *Brassicaceae* more closely resembles bud dormancy in woody perennials (Penfield, 2024), where autumn cooling induces bud set and dormancy before further winter chilling permits a state of growth receptivity to spring conditions within buds. These mechanistic parallels in phenology are significant as they suggest that floral buds and the IM remain responsive to temperature signals over the winter.

Temperature sensitivity of initiated floral buds is clear in winter oilseed rape, whereby transient warming in short days results in a subsequent delay in flowering the following spring (Lu *et al*., 2022), as opposed to an acceleration of further reproductive development through the thermosensory pathway as previous models would have suggested (Blázquez *et al*., 2003; Kumar *et al*., 2012; Posé *et al*., 2013). This delay was attributed to the up-regulation of late-vernalizing copies of *Bna.FLC* and the activation of a BRANCHED1 (BRC1)-dependent and abscisic acid (ABA)-related dormancy programme within apical buds in winter accessions (Lu *et al*., 2022). In addition to delayed flowering, warming of quiescent floral primordia in the winter also has significant effects on floral organogenesis, with observations of precocious opening of apetalous floral buds and a reduction in seed number per pod in warmed plants (Lu *et al*., 2022). This is consistent with studies which correlated warm winter temperatures with low on-farm yields (He *et al*., 2017; Brown *et al*., 2019), highlighting the importance of temperature signal integration in floral primordia for modulating yield components of crop species, although further work is necessary to better understand the mechanisms downstream of warming-induced dormancy activation which leads to yield decline.

Unlike winter annuals, mechanisms of temperature signal integration in floral buds of woody perennials have been well characterized. In aspen (*Populus* sp.), shortening photoperiods and mild cooling in autumn promote ABA accumulation in buds and an up-regulation of *SVP* orthologues (Singh *et al*., 2018, 2019). SVP subsequently promotes dormancy through up-regulation of *CALLOSE SYNTHASE 1* (*CALS1*), an established regulator of plasmodesmatal closure and symplastic isolation characteristic of dormant buds (Rinne *et al*., 2011; Tylewicz *et al*., 2018), and suppression of GA synthesis and signalling (Singh *et al*., 2018, 2019). As SVP also represses GA biosynthesis in Arabidopsis (Andrés *et al*., 2014), this suggests that mechanisms underpinning regulation of floral bud development in response to autumn and winter temperatures are conserved beyond the *Brassicaceae*.

In addition to SVP, further regulators of perennial bud dormancy are likely to have conserved roles in modulating development of floral primordia in winter annuals. DORMANCY ASSOCIATED MADS-BOX (DAM) transcription factors are master regulators of bud dormancy which are closely related to Arabidopsis *SVP* and *AGL24* (Falavigna *et al*., 2019; Quesada-Traver *et al*., 2022). Their role in regulating development of floral buds was first studied by Bielenberg *et al*. (2008), who mapped the locus responsible for constant meristematic growth in the *evergrowing* peach mutant. *DAM* gene expression is highly seasonal, responding to both temperature and photoperiod signals to control chilling responses and dormancy release. Their expression peaks in autumn and winter, inducing a dormancy state within buds, before chilling-induced epigenetic silencing which permits budbreak in spring (Leida *et al*., 2012; H. Zhu *et al*., 2020). *DAM* gene expression has been shown to control flowering time in species such as peach, apple, and loquat (Leida *et al*., 2012; Wu *et al*., 2017; Quesada-Traver *et al*., 2022), suggesting that their homologues in winter annuals might be important additional factors for controlling timing of dormancy release and growth receptivity to spring conditions. In fact, Arabidopsis SVP and AGL24 directly interact with floral integrators such as SOC1, and also repress expression of floral homeotic genes to maintain FM identity in the early stages of floral primordia development (Liu *et al*., 2008; Gregis *et al*., 2009). In the field, several *B. napus SVP* and *AGL24* genes follow expression profiles comparable with that of *DAM* genes (O’Neill *et al*., 2019), which further suggests a role for these transcription factors and their relatives in the integration of seasonal temperature cues to control development of floral buds across plant families.

## Preventing vegetative reversion

Timing the floral transition to match suitable conditions is essential to ensure reproductive success, but merely initiating an IM is not enough. Rather, the appropriate genetic machinery must be activated to ensure that the meristem remains committed to a reproductive fate (Corbesier *et al*., 2007; R. Wang *et al*., 2009; Lazaro *et al*., 2018). Reproductive commitment relies on a pair of MADS-box transcription factors which are up-regulated in the inflorescence during floral transition, *SOC1* and *FUL* (Melzer *et al*., 2008; Torti *et al*., 2012; Lazaro *et al*., 2018). Both are downstream targets of FT and therefore are under temperature control (Samach *et al*., 2000; Schmid *et al*., 2003; Teper-Bamnolker and Samach, 2005; Searle *et al*., 2006; Fig. 2). Besides their direct control by FT, both share common regulators from the thermosensory pathway such as FLM (Lee *et al*., 2013), SVP (Lee *et al*., 2007; Fujiwara *et al*., 2008; Li *et al*., 2008; Jang *et al*., 2009; Immink *et al*., 2012), and FLC (Hepworth *et al*., 2002; Helliwell *et al*., 2006; Searle *et al*., 2006; Li *et al*., 2008), which provides a local input for temperature regulation at the inflorescence level. However, unlike *FUL*, *SOC1* expression is gradually down-regulated in the absence of a floral stimulus (Torti *et al*., 2012). SOC1 and FUL form homo- and heterodimers that bind to and activate downstream genes (de Folter *et al*., 2005; Immink *et al*., 2012; Balanzà *et al*., 2014). Members of the *SPL* family have been proposed as possible targets in the context of IM commitment, and both *SOC1* and *FUL* are required for the activation of *SPL4* in the IM (Torti *et al*., 2012). In agreement with this, *SPL15* is essential for commitment in *A. alpina* (Hyun *et al*., 2019). SPL transcription factors can, in turn, promote *SOC1* and *FUL* expression (Shikata *et al*., 2009; Yamaguchi *et al*., 2009; J.-W. Wang *et al*., 2009), involving them in a feedback loop. Indeed, the expression of *SOC1* and *FUL* is controlled through an age-dependent pathway involving SPLs and miR156 (J.-W. Wang *et al*., 2009; Kim *et al*., 2012). In addition, SOC1 and FUL dimers also bind to the regulatory regions of *SOC1*, *TEM1*, *TEM2*, *SVP*, *miR156*, and *AP2*-like genes (Immink *et al*., 2012). However, one key area requiring better understanding is the environmental regulation of IM commitment, particularly given that known regulators are shared between SOC1 and FUL, despite evidence of their differing sensitivity to the environment (Torti *et al*., 2012).

## Maintaining the inflorescence meristem

In contrast to floral primordia at the apex periphery, the inflorescence is kept undifferentiated; but how? At the genetic level, the identity of both vegetative and inflorescence meristems is marked by the homeobox transcription factor WUSCHEL (WUS), which promotes cell proliferation in the AM both before and during flowering by up-regulation of *CLAVATA3* (*CLV3*) (Mayer *et al*., 1998). The activity of WUS ensures that a pool of pluripotent stem cells is maintained in the apex, and thus *WUS* expression is essential for meristem maintenance (Laux *et al*., 1996; Mayer *et al*., 1998). *WUS* itself is negatively regulated by CLV3, leading to a negative feedback loop which confines *WUS* expression within the organizing centre of the SAM (Brand *et al*., 2000; Schoof *et al*., 2000). Additional regulators of *WUS* include members of the *FANTASTIC FOUR* (*FAF*) family which are known to reduce meristem size through *WUS* inhibition (Wahl *et al*., 2010). Using modelling approaches, it has been demonstrated that changes in meristem size may affect inflorescence development by tuning the rate of organ production (Azpeitia *et al*., 2021), suggesting that mechanisms underpinning meristem maintenance may be critical for overall plant productivity.

Though WUS is pivotal for meristem maintenance, it is not exclusive to IMs, whose identity is specified by additional factors. A key example is TERMINAL FLOWER1 (TFL1), another member of the PEBP family along with FT and TSF. Similarly to FT, TFL1 forms a transcriptional regulator complex with FD; however, unlike the FT–FD complex, TFL1–FD acts predominantly by repressing the expression of downstream targets (Ahn *et al*., 2006; Hanano and Goto, 2011; Goretti *et al*., 2020). Like *WUS*, TFL1 is expressed in the IM where it inhibits the expression of FM identity genes such as *AP1* and *LFY*, therefore maintaining the identity of the indeterminate inflorescence (Gustafson-Brown *et al*., 1994; Mandel and Yanofsky, 1995; Y. Zhu *et al*., 2020). Interestingly, AP1 and LFY themselves also regulate *TFL1* expression (Goslin *et al*., 2017; Serrano-Mislata *et al*., 2017). Consequently, this crosstalk leads to spatially distinct zonation of cells belonging to either determinate FMs or indeterminate IMs. During the initial conversion of the SAM to an IM, a phenomenon of ‘doming’ is observed. Here, the IM increases in size, particularly in height, generating a dome-shaped morphology (Kinoshita *et al*., 2020; Cerise *et al*., 2023). This process is delayed by the activity of TFL1, which is achieved through repression of genes such as *FUL*, *SEPALLATA 4* (*SEP4*), *SPL3*, and *SPL8* (Goretti *et al*., 2020; Y. Zhu *et al*., 2020; Cerise *et al*., 2023).

It is also worth noting that in addition to maintaining inflorescence identity, TFL1 also functions as a negative regulator of the initial vegetative to floral transition (Hanano and Goto, 2011), a phenomenon genetically separable from its inflorescence maintenance role (Serrano-Mislata *et al*., 2017). In other species, TFL1 orthologues also repress flowering timing, and in a temperature-responsive manner (Périlleux *et al*., 2019). In the case of *A. alpina*, *AaTLF1* expression contributes to the age dependency of meristem competence for floral transition, and its expression is down-regulated during vernalization in older plants (Wang *et al*., 2011).

Owing to the multi-faceted roles of TFL1, its activity within the IM is highly dynamic. This is thanks to complex temporal and spatial regulation of *TFL1* expression and the ability of the transcribed protein to move between cells (Conti and Bradley, 2007; Goretti *et al*., 2020; Cerise *et al*., 2023). Early in development, *TFL1* is weakly expressed below and within the lower portion of the vegetative meristem, with mRNA levels increasing as development progresses towards floral transition (Bradley *et al*., 1997; Conti and Bradley, 2007). The spatial overlap of TFL1 protein with its interacting partner FD is also stage dependent, with co-localization occurring below the SAM before floral transition and at the tips of the IM after transition, reflective of the dual role of TFL1 in the repression of floral transition in vegetative meristems and later maintenance of indeterminacy in the IM (Cerise *et al*., 2023). However, during the transitionary doming stage, TFL1 becomes more diffuse, a hypothesized requirement to transiently reduce TFL1 antagonism of FT to promote floral transition (Cerise *et al*., 2023). It is therefore clear that the activity of TFL1 is critical for inflorescence development and has a direct role in controlling overall inflorescence architecture by ensuring indeterminacy. This has been demonstrated beyond Arabidopsis in crops such as *B. napus* where *tfl1* knockouts significantly reduce plant stature and fruit production (Sriboon *et al*., 2020). Additionally, targeting of the wild currant tomato (*Solanum pimpinellifolium*) *TFL1* orthologue *SELF PRUNING* (*SP*) was used as a strategy to manipulate plant architecture for the ‘*de novo* domestication’ of an ancestral progenitor species to tomato (*S. lycopersicum*) (Zsögön *et al*., 2018), highlighting agronomic applications for the manipulation of the genetic framework underpinning inflorescence establishment and maintenance (Eshed and Lippman, 2019).

Similar to TFL1, GA signalling also has a dual and separable role in floral transitions (Yamaguchi *et al*., 2014). GAs promote the vegetative to inflorescence transition, including by inducing *LFY* expression (Wilson *et al*., 1992; Blázquez *et al*., 1998; Eriksson *et al*., 2006), but LFY itself up-regulates production of a GA-catabolizing enzyme, and the resultant local reduction in GA is required to allow SPL9 to up-regulate *AP1* expression in floral primordia (Yamaguchi *et al*., 2014). As a result, GA mutants show an increased V zone but reduced I1 zone, due to delayed initial floral progression but accelerated flower formation (Yamaguchi *et al*., 2014).

However, our understanding of temperature signal integration in the control of inflorescence indeterminacy is extremely limited. It is unclear how the inflorescence-specific roles of factors such as TFL1 and GA are affected by temperature and whether this is separable from their role in initial floral transition; but this could be an interesting hypothesis to test considering the significance of temperature signals for driving development at the IM.

## Axillary meristem identity, dormancy, and outgrowth

At one time, the floral transition in Arabidopsis was proposed to be a single event which travelled through the plant, with the different identities of rosette and cauline branches being due to apical and axillary meristems ‘catching the floral wave’ at different times. In commonly used rapid-cycling accessions of Arabidopsis (e.g. Col-0 and L*er*-1) grown under standard long-day growth conditions, this may appear to be the case (Hempel and Feldman, 1994). Instead, studies working with late-flowering plants, such as rapid cyclers grown in non-inductive short days, or with winter annual accessions, challenged this paradigm in support of a two-step phase transition model (Suh *et al*., 2003). According to the latter, the trajectory of the reproductive meristem in slower flowering plants follows two stages. First, it initiates a series of lateral inflorescences which eventually develop to form the I1 region of the shoot system (Fig. 1A) and, later, it undergoes a second transition, becoming an IM, with primordia initiated thereafter acquiring FM identity, giving rise to the I2 region (Fig. 1A) (Schultz and Haughn, 1991).

In Arabidopsis, inflorescence identity acquisition is usually followed by the bolting process: the massive increase in internode length that produces the elongated inflorescence (for a review of outgrowth processes and their regulation, see McKim, 2020). However, not all AMs achieve inflorescence identity, and not all AMs bolt, with many instead remaining very slow-growing ‘axillary buds’, a state usually termed ‘dormant’, even though they may be producing many lateral organs. Evidently, AMs have different sensitivities to flowering-promotive signals (Niwa *et al*., 2013; Lazaro *et al*., 2018; Zhu and Wagner, 2020; Fig. 4). The I1 (‘cauline’) zone is a clear case; primordia initiated during the I1 phase usually grow out rapidly at bolting, producing further inflorescences (Lazaro *et al*., 2018). In contrast I2 zone AMs are pre-committed as FMs. Thus the rate of progression from the vegetative meristem to I1 then to I2 will set the pattern of axillary identities and subsequent architecture. Indeed, it has been proposed that the difference in inflorescence architectures between Arabidopsis and other species from the *Brassicaceae* (e.g. cauliflower) is likely to be due to differences in the balance of gene activity in floral networks (Prusinkiewicz *et al*., 2007; Azpeitia *et al*., 2021).

Interacting with this floral identity pattern are other processes that control relative activity and maintenance of AMs, particularly apical dominance and resource availability. These are largely processes in which local growth is coordinated with the whole-plant body plan by movement and local synthesis of photosynthates and hormones—particularly auxin, cytokinins (CKs), and strigolactones (SLs). For detailed reviews on the mode of action of these signals, see Zhu and Wagner (2020) and Barbier *et al*. (2023). In brief, auxin produced in existing inflorescences acts to competitively inhibit the outgrowth of other AMs (Ongaro *et al*., 2008; Prusinkiewicz *et al*., 2009; Bennett *et al*., 2016). Auxin effects at the AM itself are mediated indirectly as auxin itself does not enter the bud. Auxin enhances SL synthesis, which in turn enhances the competitive effect of auxin, concentrating growth in actively elongating branches (Crawford *et al*., 2010; Bennett *et al*., 2016). SL action in the bud is partly mediated by up-regulating expression of the repressive TCP transcription factor *BRC1* (Aguilar-Martínez *et al*., 2007; Wang *et al*., 2015). Auxin also down-regulates CK production; CKs act locally in the bud to promote outgrowth at the cell cycle level (Tanaka *et al*., 2006). After dormancy release, commitment to sustained bud outgrowth is further supported by auxin export from the bud itself (Chabikwa *et al*., 2019), but also by GA signalling in a manner analogous to GA control of outgrowth of the main shoot, as reviewed elsewhere (McKim, 2020).

This hormonal network coordinates the effect of nutrient levels perceived in the roots (via modulation of SL and CK synthesis) and loss of resource sinks (auxin and apical dominance) with the local AM transcriptional programme. When dormant, AMs are characterized by a starvation-like transcriptional programme downstream of BRC1, which activates a triad of homeodomain leucine zipper (HD-Zip) transcription factors, HOMEOBOX PROTEIN21 (HB21), HB40, and HB53, to up-regulate local ABA signalling (González-Grandío *et al*., 2017; Martín-Fontecha *et al*., 2018). Photosynthate availability, communicated via trehalose-6-phosphate, counteracts this directly at the bud to promote outgrowth by promoting *FT* and *TSF* expression (Fichtner *et al*., 2021), much as it does at the whole-plant scale to promote flowering generally (Wahl *et al*., 2013). Sucrose and citrate also act to promote branching via the SL signalling pathway and CK levels (Bertheloot *et al*., 2020; Barbier *et al*., 2021; Salam *et al*., 2021; reviewed by Barbier *et al*., 2023).

Hormone levels, their respective signalling pathways, and photosynthate are all influenced by temperature (reviewed by Castroverde and Dina, 2021) although, with the exception of GA and ABA, the effects of these on flowering have largely not been studied, especially in field contexts. However, temperature-regulated floral genes such as *FLC*, *FT*, and the *SPL* family are closely interlinked to this network. *FLC* changes the pattern of branch outgrowth along the main axis, and vernalization promotes branch outgrowth in Arabidopsis and *A. alpina* (Huang *et al*., 2013; Lazaro *et al*., 2018; Vayssières *et al*., 2020). Indeed, *FLC* is one of the largest effect loci controlling branching in natural variants of Arabidopsis (Huang *et al*., 2013; Jong *et al*., 2019; Hepworth *et al*., 2020). To what extent this is a pre-patterning effect on meristem floral progression versus an effect later and local to buds is not clear, as FLC binds to the *BRC1* locus in Arabidopsis while *PEP1* does not in *A. alpina* (Mateos *et al*., 2015, 2017). However, *A. alpina* is a perennial, and reactivation of *PEP1* in meristems is important to return plants to a non-flowering state over summer and autumn (R. Wang *et al*., 2009; Lazaro *et al*., 2018). In *B. napus*, up-regulation of *FLC* copies in warm winters is associated with up-regulation of *BRC1* copies in floral buds (Lu *et al*., 2022). In Citrus trees, local patterning of the *FLC* family member *CcMADS19* is associated with local control of *FT* expression, suppressing new flower formation adjacent to forming fruits (Agustí *et al.*, 2020; Mesejo *et al*., 2022), suggesting that local *FLC* expression may be a conserved mechanism for patterning perennials over seasons.

Downstream of FLC, SPL15 and SPL9 directly repress *BRC1* expression, promoting outgrowth of the bud, but this regulation is disrupted by the SL signalling components SUPPRESSOR OF MAX2-LIKE (SMXL6/7/8) proteins (Xie *et al*., 2020), a regulatory loop first identified in rice (Lu *et al*., 2013; Song *et al*., 2017). SLs have also been identified as delaying flowering via promotion of the protein activity of the repressive AP2 family member TOE1 (Bai *et al*., 2024). SLs communicate a number of abiotic inputs to plant architecture (Castroverde and Dina, 2021): whether temperature is an important factor for SL-mediated control of branch outgrowth is not yet well studied in the *Brassicaceae*.

The florigens FT and TSF have a direct local promotive effect on branch outgrowth, moving into buds via the vasculature from the subtending leaf to interact with BRC1 at the protein level and promote outgrowth (Huang *et al*., 2013; Niwa *et al*., 2013). BRC1 presence in AMs reduces expression of *SOC1*, *AP1*, and *FUL*, and, if misexpressed at the main SAM, delays flowering in Arabidopsis (Niwa *et al*., 2013). Thus the activities of BRC1 and FT are opposing for both bud outgrowth and floral identity, an effect conserved between Arabidopsis and wheat (Niwa *et al*., 2013; Dixon *et al*., 2018). Indeed, in poplar, the opposing action of BRC1 and FT orthologues is key to seasonal dormancy, with short day-induced growth cessation and bud set in autumn involving the down-regulation of PtFT1 and up-regulation of *BRC1* (Böhlenius *et al*., 2006; Singh *et al*., 2018). As noted previously (‘Floral buds are winter structures’), SVP-like genes are also involved in poplar seasonal dormancy, further enhancing the links between seasonal cessation of growth and reproductive identity of meristems.

## The end of the road: inflorescence arrest

Just like the initiation of inflorescences, and of organs therein, growth arrest of the reproductive structures is a key determinant of reproductive output. Indeed, plants do not flower indefinitely, rather inflorescences tend to interrupt their development after producing a variable number of fruits through a process termed proliferative arrest (Hensel *et al*., 1994). In Arabidopsis, two key phenomena are needed for this. On the one hand, the inflorescence must cease to initiate new floral primordia through IM arrest. On the other hand, not all of the initiated primordia complete floral development such that some of the youngest floral buds arrest their own development through the process of floral arrest.

At its core, IM arrest induces a reversible inactivation of the meristematic activity, coupled with a transcriptional down-regulation of the meristem maintenance integrator *WUS* (Balanzà *et al*., 2018; Merelo *et al*., 2022). This is under strong regulation by endogenous signals, particularly age. In older plants, higher levels of miR172 induce a transcriptional repression of *AP2*, a known activator of *WUS*. The environmental control of inflorescence arrest has been less investigated, but recent work has identified a role for temperature in regulating this process (Miryeganeh, 2020; González-Suárez *et al*., 2023), adding to a growing body of literature which supports that warming accelerates the end of flowering in various *Brassicaceae* (Lomas and Burd, 1983; Satake *et al*., 2013; Cao *et al*., 2016; Nagahama *et al*., 2018; Komoto *et al*., 2024). The molecular basis of this phenomenon is still not fully understood, but an attractive hypothesis is that temperature integration takes place upstream of *FT*, similar to what has been observed for the onset of flowering. After floral transition, *FT* is up-regulated over time in a temperature-dependent manner, and this seems necessary to trigger timely inflorescence arrest (González-Suárez *et al*., 2023). Within this model, how temperature feeds into *FT* expression is not well understood, but it is possible that this is through MADS box transcription factor complexes such as SVP–FLM and SVP–FLC, as knockout of these genes disrupts the response to temperature (González-Suárez *et al*., 2023). More intriguing is how a floral promotive signal such as FT can act to shut down the proliferative activity of the IM, which still remains an open question. In addition to upstream regulation of *FT*, another possibility is that temperature inputs directly into the age pathway, as miR172 accumulation is also induced in the warmth by known thermosensory regulators, such as FCA, SVP, and FLM (see ‘The making of inflorescence meristems’).

In the field, temperature control of inflorescence arrest is even less well characterized. However, perennial relatives of Arabidopsis show remarkable seasonal changes in their transcriptome, particularly in genes implicated in flowering (Nagano *et al*., 2019; Komoto *et al*., 2024). The end of the reproductive season has been associated with a peak of *FT* expression, which sharply declines thereafter and remains off until the next spring (Satake *et al*., 2013; Fig. 3B). In turn, *FLC* expression is gradually recovered during spring concomitant with an increase in temperatures, leading to its complete reactivation by the end of the flowering period. Both of these are highly temperature dependent. Interestingly, warming seems to accelerate the shutdown of *FT* to a greater degree than its activation and, as such, it shortens the duration of flowering (Satake *et al*., 2013). Several targets have been proposed to mediate this, including *SOC1*, GA synthesis genes, and members of the age pathway such as *SPL15*, all of which are negatively regulated by *FLC* and seasonally fluctuating (Komoto *et al*., 2024; Fig. 3B), but their specific function in inflorescence arrest is unclear. To better understand this, future research would benefit from extending lab studies to more realistic temperature scenarios using winter accessions of Arabidopsis, particularly considering the central role of *FLC* in promoting arrest in the *Brassicaceae* family (R. Wang *et al*., 2009; Satake *et al*., 2013).

Even after inflorescence arrest, already-initiated primordia can continue to develop and open into flowers. Thus, towards the end of the reproductive phase, plants must halt development of incipient floral primordia to focus their energy investment on pod filling, maximizing the reproductive potential of established fruits before the onset of autumn. It is important to distinguish floral arrest from FM termination, which marks the end of floral organogenesis, leading to a complete whorl of sepals, petals, stamens, and carpels (Min and Kramer, 2023). Instead, floral arrest takes place after all of the floral organs have been specified, and results in the distinctive cluster of undeveloped buds typical of arrested inflorescences (Hensel *et al*., 1994; Walker *et al*., 2023). Recently, this has been shown to be regulated by the same triad of HD-Zip TFs that are regulated by BRC1, and which promote ABA accumulation in floral buds during arrest in a manner similar to dormant axillary buds (Sánchez-Gerschon *et al*., 2024). ABA has also been implicated in delaying development of *B. napus* floral primordia in response to warm temperatures through up-regulation of *BRC1*, *HB21*, *HB40*, *HB53*, and ABA biosynthesis genes such as *NINE-CIS-EPOXYCAROTENOID DIOXYGENASE-3* (*NCED3*) (Lu *et al*., 2022). This suggests that ABA might act as a general signal to block development of floral buds throughout the lifetime of the inflorescence, and that this inhibitory mechanism can be modulated by temperature signals (see ‘Floral buds are winter structures’). Nevertheless, additional signals are likely to be important for promoting floral bud arrest, as otherwise both newly initiated floral primordia and dormant axillary buds would arrest under certain conditions if ABA-related regulatory modules acted alone. Promising candidates for this are auxin and CK, which show promotive and inhibitory effects on floral bud arrest, respectively (Ware *et al*., 2020; Walker *et al*., 2023). Temperature sensitivity at the level of *FT* expression is key for timely IM arrest (González-Suárez *et al*., 2023), and this sensitivity has also been observed throughout inflorescence development in younger siliques (González-Suárez *et al*., 2024), posing the question of whether floral bud arrest may be under similar environmental control, though this remains to be investigated.

## Conclusions

IMs are transient structures whose development is critical for the reproductive potential of all flowering plants. These tissues can be present throughout the reproductive phase and therefore experience a wide range of both environmental and endogenous signals, often interpreting these simultaneously to coordinate adaptive growth. Considering the significance of plant reproductive tissues for global agriculture and food security, understanding how the inflorescence responds to environmental signals at different developmental stages will be essential for unravelling the effects of climate change on crops. However, relatively little work has been done on the integration of temperature with later stages of meristem development, especially in the critical context of the field (Fig. 4). The baseline for our knowledge of the genetics of inflorescence formation mostly derives from the standard Arabidopsis growth conditions (long daylength >14 h, and warmer than 18 °C day and night); conditions which are at best loosely representative of late spring and high summer in most temperate regions. Within Arabidopsis, the genotypes more frequently studied are (with notable exceptions) rapid cyclers, such that flowering is highly accelerated in these conditions. It is perhaps unsurprising then that many recent advances in inflorescence progression and shoot architecture patterning derive from perennial relatives of Arabidopsis, and crops such as *Brassica*, poplar, and fruit trees, which have to be studied in longer lived contexts. Nevertheless, many important questions still remain, several of which regard the balance of effects at different temperatures and times of year. While research has uncovered many conserved pathways controlling seasonal inflorescence growth, for crop breeding details matter. Precise knowledge of temperature ranges at which the various genetic pathways act in the field, and the result of changing these by 2–3 °C, is currently lacking. Given current global warming trends, it is urgently needed.

## Data Availability

No new data were generated or analysed in support of this research. Free to use, editable versions of all figures and diagrams can be found at http://www.pablidopsis.com/resources.

## Abbreviations

ABA: abscisic acid
AGL24: AGAMOUS-LIKE24
AM: axillary meristem
AP1: APETALA1
AP2: APETALA2
AP2L: APETALA2-LIKE
BRC1: BRANCHED1
CK: cytokinin
CLV3: CLAVATA3
DAM: DORMANCY ASSOCIATED MADS-BOX
ELF: EARLY FLOWERING
FCA: FLOWERING CONTROL LOCUS A
FLC: FLOWERING LOCUS C
FLM: FLOWERING LOCUS M
FM: floral meristem
FRI: FRIGIDA
FT: FLOWERING LOCUS T
FUL: FRUITFUL
GA: gibberellin
GA20OX: GIBBERELLIN 20-OXIDASE
GA3OX: GIBBERELLIN 3-OXIDASE
GAI: GA INSENSITIVE
HB: HOMEOBOX PROTEIN
HD-Zip: homeodomain leucine zipper
I1/2: inflorescence zone 1/2
IM: inflorescence meristem
LFY: LEAFY
MAF: MADS AFFECTING FLOWERING
PEBP: Phosphatidylethanolamine -binding protein
PEP1: PERPETUAL FLOWERING1
PIF: PHYTOCHROME INTERACTING FACTOR
PWR: POWERDRESS
RGA: REPRESSOR OF GAI
RGL: RGA-LIKE
SAM: shoot apical meristem
SL: strigolactone
SOC1: SUPPRESSOR OF OVEREXPRESSION OF CONSTANS1
SPL: SQUAMOSA PROMOTER BINDING-LIKE
SVP: SHORT VEGETATIVE PHASE
TCP: TEOSINTE BRANCHED1/CYCLOIDEA/PCF
TEM: TEMPRANILLO
TFL1: TERMINAL FLOWER1
TOE: TARGET OF EAT
TSF: TWIN SISTER OF FT
V1/2/3: vegetative zone 1–3
VIN3: VERNALIZATION INSENSITIVE3
VM: vegetative SAM
WUS: WUSCHEL

## References

[eraf261-B1] Abe M, Kobayashi Y, Yamamoto S, Daimon Y, Yamaguchi A, Ikeda Y, Ichinoki H, Notaguchi M, Goto K, Araki T. 2005. FD, a bZIP protein mediating signals from the floral pathway integrator FT at the shoot apex. Science 309, 1052–1056. 10.1126/science.1115983 16099979

[eraf261-B2] Ågren J, Oakley CG, Lundemo S, Schemske DW. 2017. Adaptive divergence in flowering time among natural populations of Arabidopsis thaliana: estimates of selection and QTL mapping. Evolution 71, 550–564. 10.1111/evo.13126 27859214

[eraf261-B3] Aguilar-Martínez JA, Poza-Carrión C, Cubas P. 2007. Arabidopsis BRANCHED1 acts as an integrator of branching signals within axillary buds. The Plant Cell 19, 458–472. 17307924 10.1105/tpc.106.048934 PMC1867329

[eraf261-B4] Agustí M, Mesejo C, Muñoz-Fambuena N, Vera-Sirera F, de Lucas M, Martínez-Fuentes A, Reig C, Iglesias DJ, Primo-Millo E, Blázquez MA. 2020. Fruit-dependent epigenetic regulation of flowering in Citrus. New Phytologist 225, 376–384. 31273802 10.1111/nph.16044

[eraf261-B5] Ahn JH, Miller D, Winter VJ, Banfield MJ, Lee JH, Yoo SY, Henz SR, Brady RL, Weigel D. 2006. A divergent external loop confers antagonistic activity on floral regulators FT and TFL1. The EMBO Journal 25, 605–614. 16424903 10.1038/sj.emboj.7600950 PMC1383534

[eraf261-B6] Alemán-Báez J, Qin J, Cai C, Zou C, Bucher J, Paulo M-J, Voorrips RE, Bonnema G. 2022. Genetic dissection of morphological variation in rosette leaves and leafy heads in cabbage (*Brassica oleracea* var*. capitata*). Theoretical and Applied Genetics 135, 3611–3628. 10.1007/s00122-022-04205-w PMC9519658 36057748

[eraf261-B7] Andrés F, Porri A, Torti S, Mateos J, Romera-Branchat M, García-Martínez JL, Fornara F, Gregis V, Kater MM, Coupland G. 2014. SHORT VEGETATIVE PHASE reduces gibberellin biosynthesis at the Arabidopsis shoot apex to regulate the floral transition. Proceedings of the National Academy of Sciences, USA 111, E2760–E2769. 10.1073/pnas.1409567111 PMC4084417 24979809

[eraf261-B8] Antoniou-Kourounioti RL, Hepworth J, Heckmann A, Duncan S, Qüesta J, Rosa S, Säll T, Holm S, Dean C, Howard M. 2018. Temperature sensing is distributed throughout the regulatory network that controls *FLC* epigenetic silencing in vernalization. Cell Systems 7, 643–655.e9. 30503646 10.1016/j.cels.2018.10.011 PMC6310686

[eraf261-B9] Aukerman MJ, Sakai H. 2003. Regulation of flowering time and floral organ identity by a MicroRNA and its *APETALA2-like* target genes. The Plant Cell 15, 2730–2741. 14555699 10.1105/tpc.016238 PMC280575

[eraf261-B10] Azpeitia E, Tichtinsky G, Le Masson M, et al 2021. Cauliflower fractal forms arise from perturbations of floral gene networks. Science 373, 192–197. 10.1126/science.abg5999 34244409

[eraf261-B11] Bai J, Lei X, Liu J, et al 2024. The strigolactone receptor DWARF14 regulates flowering time in Arabidopsis. The Plant Cell 36, 4752–4767. 39235115 10.1093/plcell/koae248 PMC11530773

[eraf261-B12] Balanzà V, Martínez-Fernández I, Ferrándiz C. 2014. Sequential action of FRUITFULL as a modulator of the activity of the floral regulators SVP and SOC1. Journal of Experimental Botany 65, 1193–1203. 10.1093/jxb/ert482 PMC3935574 24465009

[eraf261-B13] Balanzà V, Martínez-Fernández I, Sato S, Yanofsky MF, Kaufmann K, Angenent GC, Bemer M, Ferrándiz C. 2018. Genetic control of meristem arrest and life span in Arabidopsis by a FRUITFULL-APETALA2 pathway. Nature Communications 9, 565. 10.1038/s41467-018-03067-5 PMC5805735 29422669

[eraf261-B14] Balasubramanian S, Sureshkumar S, Lempe J, Weigel D. 2006. Potent induction of *Arabidopsis thaliana* flowering by elevated growth temperature. PLOS Genetics 2, e106. 16839183 10.1371/journal.pgen.0020106 PMC1487179

[eraf261-B15] Barbier FF, Cao D, Fichtner F, Weiste C, Perez-Garcia MD, Caradeuc M, Le Gourrierec J, Sakr S, Beveridge CA. 2021. HEXOKINASE1 signalling promotes shoot branching and interacts with cytokinin and strigolactone pathways. New Phytologist 231, 1088–1104. 33909299 10.1111/nph.17427

[eraf261-B16] Barbier F, Fichtner F, Beveridge C. 2023. The strigolactone pathway plays a crucial role in integrating metabolic and nutritional signals in plants. Nature Plants 9, 1191–1200. 37488268 10.1038/s41477-023-01453-6

[eraf261-B17] Benlloch R, Berbel A, Serrano-Mislata A, Madueño F. 2007. Floral initiation and inflorescence architecture: a comparative view. Annals of Botany 100, 659–676. 17679690 10.1093/aob/mcm146 PMC2533596

[eraf261-B18] Bennett T, Hines G, van Rongen M., Waldie T, Sawchuk MG, Scarpella E, Ljung K, Leyser O. 2016. Connective auxin transport in the shoot facilitates communication between shoot apices. PLOS Biology 14, e1002446. 10.1371/journal.pbio.1002446 PMC4847802 27119525

[eraf261-B19] Bertheloot J, Barbier F, Boudon F, Perez-Garcia MD, Péron T, Citerne S, Dun E, Beveridge C, Godin C, Sakr S. 2020. Sugar availability suppresses the auxin-induced strigolactone pathway to promote bud outgrowth. New Phytologist 225, 866–879. 31529696 10.1111/nph.16201

[eraf261-B20] Bielenberg DG, Wang YE, Li Z, Zhebentyayeva T, Fan S, Reighard GL, Scorza R, Abbott AG. 2008. Sequencing and annotation of the evergrowing locus in peach [*Prunus persica* (L.) Batsch] reveals a cluster of six MADS-box transcription factors as candidate genes for regulation of terminal bud formation. Tree Genetics & Genomes 4, 495–507.

[eraf261-B21] Blázquez MA, Ahn JH, Weigel D. 2003. A thermosensory pathway controlling flowering time in Arabidopsis thaliana. Nature Genetics 33, 168–171. 10.1038/ng1085 12548286

[eraf261-B22] Blázquez MA, Green R, Nilsson O, Sussman MR, Weigel D. 1998. Gibberellins promote flowering of Arabidopsis by activating the LEAFY promoter. The Plant Cell 10, 791–800. 9596637 10.1105/tpc.10.5.791 PMC144373

[eraf261-B23] Böhlenius H, Huang T, Charbonnel-Campaa L, Brunner AM, Jansson S, Strauss SH, Nilsson O. 2006. CO/FT regulatory module controls timing of flowering and seasonal growth cessation in trees. Science 312, 1040–1043. 16675663 10.1126/science.1126038

[eraf261-B24] Bond DM, Dennis ES, Pogson BJ, Finnegan EJ. 2009. Histone acetylation, VERNALIZATION INSENSITIVE 3, FLOWERING LOCUS C, and the vernalization response. Molecular Plant 2, 724–737. 19825652 10.1093/mp/ssp021

[eraf261-B25] Bradley D, Ratcliffe O, Vincent C, Carpenter R, Coen E. 1997. Inflorescence commitment and architecture in Arabidopsis. Science 275, 80–83. 8974397 10.1126/science.275.5296.80

[eraf261-B26] Brand U, Fletcher JC, Hobe M, Meyerowitz EM, Simon R. 2000. Dependence of stem cell fate in Arabidopsis on a feedback loop regulated by CLV3 activity. Science 289, 617–619. 10915624 10.1126/science.289.5479.617

[eraf261-B27] Brown JKM, Beeby R, Penfield S. 2019. Yield instability of winter oilseed rape modulated by early winter temperature. Scientific Reports 9, 6953. 10.1038/s41598-019-43461-7 PMC6502865 31061437

[eraf261-B28] Cao Y, Xiao Y, Huang H, Xu J, Hu W, Wang N. 2016. Simulated warming shifts the flowering phenology and sexual reproduction of *Cardamine hirsuta* under different planting densities. Scientific Reports 6, 27835. 27296893 10.1038/srep27835 PMC4906517

[eraf261-B29] Castillejo C, Pelaz S. 2008. The balance between CONSTANS and TEMPRANILLO activities determines *FT* expression to trigger flowering. Current Biology 18, 1338–1343. 10.1016/j.cub.2008.07.075 18718758

[eraf261-B30] Castroverde CDM, Dina D. 2021. Temperature regulation of plant hormone signaling during stress and development. Journal of Experimental Botany 72, 7436–7458. 10.1093/jxb/erab257 34081133

[eraf261-B31] Cerise M, da Silveira Falavigna V, Rodríguez-Maroto G, et al 2023. Two modes of gene regulation by TFL1 mediate its dual function in flowering time and shoot determinacy of Arabidopsis. Development 150, dev202089. 10.1242/dev.202089 PMC10730086 37971083

[eraf261-B32] Chabikwa TG, Brewer PB, Beveridge CA. 2019. Initial bud outgrowth occurs independent of auxin flow from out of buds. Plant Physiology 179, 55–65. 10.1104/pp.18.00519 PMC6324225 30404820

[eraf261-B34] Chen Q, Payyavula RS, Chen L, Zhang J, Zhang C, Turgeon R. 2018. *FLOWERING LOCUS T* mRNA is synthesized in specialized companion cells in *Arabidopsis* and Maryland mammoth tobacco leaf veins. Proceedings of the National Academy of Sciences, USA 115, 2830–2835. 10.1073/pnas.1719455115 PMC5856545 29483267

[eraf261-B33] Chen X . 2004. A MicroRNA as a translational repressor of APETALA2 in Arabidopsis flower development. Science 303, 2022–2025. 12893888 10.1126/science.1088060 PMC5127708

[eraf261-B35] Cheng H, Qin L, Lee S, Fu X, Richards DE, Cao D, Luo D, Harberd NP, Peng J. 2004. Gibberellin regulates Arabidopsis floral development via suppression of DELLA protein function. Development 131, 1055–1064. 14973286 10.1242/dev.00992

[eraf261-B36] Cheng Y-J, Shang G-D, Xu Z-G, Yu S, Wu L-Y, Zhai D, Tian S-L, Gao J, Wang L, Wang J-W. 2021. Cell division in the shoot apical meristem is a trigger for miR156 decline and vegetative phase transition in Arabidopsis. Proceedings of the National Academy of Sciences, USA 118, e2115667118. 10.1073/pnas.2115667118 PMC8609562 34750273

[eraf261-B37] Cho HJ, Kim JJ, Lee JH, Kim W, Jung J-H, Park C-M, Ahn JH. 2012. SHORT VEGETATIVE PHASE (SVP) protein negatively regulates miR172 transcription via direct binding to the pri-miR172a promoter in Arabidopsis. FEBS Letters 586, 2332–2337. 22659182 10.1016/j.febslet.2012.05.035

[eraf261-B38] Conti L, Bradley D. 2007. TERMINAL FLOWER1 is a Mobile signal controlling Arabidopsis architecture. The Plant Cell 19, 767–778. 10.1105/tpc.106.049767 PMC1867375 17369370

[eraf261-B39] Corbesier L, Vincent C, Jang S, et al 2007. FT protein movement contributes to long-distance signaling in floral induction of Arabidopsis. Science 316, 1030–1033. 17446353 10.1126/science.1141752

[eraf261-B40] Crawford S, Shinohara N, Sieberer T, Williamson L, George G, Hepworth J, Müller D, Domagalska MA, Leyser O. 2010. Strigolactones enhance competition between shoot branches by dampening auxin transport. Development 137, 2905–2913. 20667910 10.1242/dev.051987

[eraf261-B41] de Folter S, Immink RGH, Kieffer M, et al 2005. Comprehensive interaction map of the Arabidopsis MADS box transcription factors. The Plant Cell 17, 1424–1433. 10.1105/tpc.105.031831 PMC1091765 15805477

[eraf261-B42] de Lucas M, Davière J-M, Rodríguez-Falcón M, Pontin M, Iglesias-Pedraz JM, Lorrain S, Fankhauser C, Blázquez MA, Titarenko E, Prat S. 2008. A molecular framework for light and gibberellin control of cell elongation. Nature 451, 480–484. 18216857 10.1038/nature06520

[eraf261-B43] De Lucia F, Crevillen P, Jones AME, Greb T, Dean C. 2008. A PHD-Polycomb Repressive Complex 2 triggers the epigenetic silencing of *FLC* during vernalization. Proceedings of the National Academy of Sciences, USA 105, 16831–16836. 10.1073/pnas.0808687105 PMC2579339 18854416

[eraf261-B44] Dixon LE, Greenwood JR, Bencivenga S, Zhang P, Cockram J, Mellers G, Ramm K, Cavanagh C, Swain SM, Boden SA. 2018. TEOSINTE BRANCHED1 regulates inflorescence architecture and development in bread wheat (*Triticum aestivum*). The Plant Cell 30, 563–581. 29444813 10.1105/tpc.17.00961 PMC5894836

[eraf261-B45] Dixon LE, Karsai I, Kiss T, Adamski NM, Liu Z, Ding Y, Allard V, Boden SA, Griffiths S. 2019. VERNALIZATION1 controls developmental responses of winter wheat under high ambient temperatures. Development 146, dev172684. 10.1242/dev.172684 PMC6382010 30770359

[eraf261-B46] Duncan S, Holm S, Questa J, Irwin J, Grant A, Dean C. 2015. Seasonal shift in timing of vernalization as an adaptation to extreme winter. eLife 4, e06620. 26203563 10.7554/eLife.06620 PMC4532801

[eraf261-B47] Eriksson S, Böhlenius H, Moritz T, Nilsson O. 2006. GA4 is the active gibberellin in the regulation of LEAFY transcription and Arabidopsis floral initiation. The Plant Cell 18, 2172–2181. 16920780 10.1105/tpc.106.042317 PMC1560906

[eraf261-B48] Eshed Y, Lippman ZB. 2019. Revolutions in agriculture chart a course for targeted breeding of old and new crops. Science 366, eaax0025. 10.1126/science.aax0025 31488704

[eraf261-B49] Ezer D, Jung J-H, Lan H, et al 2017. The evening complex coordinates environmental and endogenous signals in Arabidopsis. Nature Plants 3, 17087. 28650433 10.1038/nplants.2017.87 PMC5495178

[eraf261-B50] Falavigna VDS, Guitton B, Costes E, Andrés F. 2019. I want to (bud) break free: the potential role of DAM and SVP-like genes in regulating dormancy cycle in temperate fruit trees. Frontiers in Plant Science 9, 1990. 30687377 10.3389/fpls.2018.01990 PMC6335348

[eraf261-B51] Fichtner F, Barbier FF, Annunziata MG, Feil R, Olas JJ, Mueller-Roeber B, Stitt M, Beveridge CA, Lunn JE. 2021. Regulation of shoot branching in arabidopsis by trehalose 6-phosphate. New Phytologist 229, 2135–2151. 10.1111/nph.17006 33068448

[eraf261-B52] Fujiwara S, Oda A, Yoshida R, et al 2008. Circadian clock proteins LHY and CCA1 regulate SVP protein accumulation to control flowering in Arabidopsis. The Plant Cell 20, 2960–2971. 19011118 10.1105/tpc.108.061531 PMC2613671

[eraf261-B53] Gandikota M, Birkenbihl RP, Höhmann S, Cardon GH, Saedler H, Huijser P. 2007. The miRNA156/157 recognition element in the 3’ UTR of the Arabidopsis SBP box gene *SPL3* prevents early flowering by translational inhibition in seedlings. The Plant Journal 49, 683–693. 17217458 10.1111/j.1365-313X.2006.02983.x

[eraf261-B54] Gazzani S, Gendall AR, Lister C, Dean C. 2003. Analysis of the molecular basis of flowering time variation in Arabidopsis accessions. Plant Physiology 132, 1107–1114. 12805638 10.1104/pp.103.021212 PMC167048

[eraf261-B55] González-Grandío E, Pajoro A, Franco-Zorrilla JM, Tarancón C, Immink RGH, Cubas P. 2017. Abscisic acid signaling is controlled by a *BRANCHED1/HD-ZIP I* cascade in *Arabidopsis* axillary buds. Proceedings of the National Academy of Sciences, USA 114, E245–E254. 10.1073/pnas.1613199114 PMC5240681 28028241

[eraf261-B56] González-Suárez P, Walker CH, Bennett T. 2023. FLOWERING LOCUS T mediates photo-thermal timing of inflorescence meristem arrest in Arabidopsis thaliana. Plant Physiology 192, 2276–2289. 36943252 10.1093/plphys/kiad163 PMC10315265

[eraf261-B57] González-Suárez P, Walker CH, Lock T, Bennett T. 2024. FLOWERING LOCUS T-mediated thermal signalling regulates age-dependent inflorescence development in Arabidopsis thaliana. Journal of Experimental Botany 75, 4400–4414. 10.1093/jxb/erae094 PMC11263484 38442244

[eraf261-B58] Goretti D, Silvestre M, Collani S, Langenecker T, Méndez C, Madueño F, Schmid M. 2020. TERMINAL FLOWER1 functions as a mobile transcriptional cofactor in the shoot apical meristem. Plant Physiology 182, 2081–2095. 31996406 10.1104/pp.19.00867 PMC7140938

[eraf261-B59] Goslin K, Zheng B, Serrano-Mislata A, et al 2017. Transcription factor interplay between LEAFY and APETALA1/CAULIFLOWER during floral initiation. Plant Physiology 174, 1097–1109. 28385730 10.1104/pp.17.00098 PMC5462026

[eraf261-B60] Gray AR . 1982. Taxonomy and evolution of broccoli (*Brassica oleracea* var. *italica*). Economic Botany 36, 397–410.

[eraf261-B61] Gregis V, Sessa A, Dorca-Fornell C, Kater MM. 2009. The Arabidopsis floral meristem identity genes AP1, AGL24 and SVP directly repress class B and C floral homeotic genes. The Plant Journal 60, 626–637. 10.1111/j.1365-313X.2009.03985.x 19656343

[eraf261-B62] Griffiths J, Murase K, Rieu I, et al 2006. Genetic characterization and functional analysis of the GID1 gibberellin receptors in Arabidopsis. The Plant Cell 18, 3399–3414. 17194763 10.1105/tpc.106.047415 PMC1785415

[eraf261-B63] Gu X, Le C, Wang Y, Li Z, Jiang D, Wang Y, He Y. 2013. Arabidopsis FLC clade members form flowering-repressor complexes coordinating responses to endogenous and environmental cues. Nature Communications 4, 1947. 10.1038/ncomms2947 PMC3709509 23770815

[eraf261-B64] Gustafson-Brown C, Savidge B, Yanofsky MF. 1994. Regulation of the arabidopsis floral homeotic gene *APETALA1*. Cell 76, 131–143. 10.1016/0092-8674(94)90178-3 7506995

[eraf261-B65] Han X, Yu H, Yuan R, Yang Y, An F, Qin G. 2019. Arabidopsis transcription factor TCP5 controls plant thermomorphogenesis by positively regulating PIF4 activity. iScience 15, 611–622. 31078552 10.1016/j.isci.2019.04.005 PMC6548983

[eraf261-B66] Hanano S, Goto K. 2011. Arabidopsis TERMINAL FLOWER1 is involved in the regulation of flowering time and inflorescence development through transcriptional repression. The Plant Cell 23, 3172–3184. 21890645 10.1105/tpc.111.088641 PMC3203435

[eraf261-B67] He Y, Revell BJ, Leng B, Feng Z. 2017. The effects of weather on oilseed rape (OSR) yield in China: future implications of climate change. Sustainability 9, 418.

[eraf261-B68] Hedden P . 2020. The Current Status of Research on Gibberellin Biosynthesis. Plant & Cell Physiology 61, 1832–1849. 32652020 10.1093/pcp/pcaa092 PMC7758035

[eraf261-B69] Heisler MG, Jönsson H, Wenkel S, Kaufmann K. 2022. Context-specific functions of transcription factors controlling plant development: from leaves to flowers. Current Opinion in Plant Biology 69, 102262. 35952407 10.1016/j.pbi.2022.102262

[eraf261-B70] Helliwell CA, Wood CC, Robertson M, James Peacock W, Dennis ES. 2006. The Arabidopsis FLC protein interacts directly in vivo with *SOC1* and *FT* chromatin and is part of a high-molecular-weight protein complex. The Plant Journal 46, 183–192. 10.1111/j.1365-313X.2006.02686.x 16623882

[eraf261-B71] Hempel FD, Feldman LJ. 1994. Bi-directional inflorescence development in Arabidopsis thaliana: acropetal initiation of flowers and basipetal initiation of paraclades. Planta 192, 276–286.

[eraf261-B72] Hensel LL, Nelson MA, Richmond TA, Bleecker AB. 1994. The fate of inflorescence meristems is controlled by developing fruits in Arabidopsis. Plant Physiology 106, 863–876. 10.1104/pp.106.3.863 PMC159609 7824655

[eraf261-B73] Hepworth J, Antoniou-Kourounioti RL, Berggren K, et al 2020. Natural variation in autumn expression is the major adaptive determinant distinguishing Arabidopsis *FLC* haplotypes. Elife 9, e57671. 10.7554/eLife.57671 PMC7518893 32902380

[eraf261-B74] Hepworth J, Antoniou-Kourounioti RL, Bloomer RH, et al 2018. Absence of warmth permits epigenetic memory of winter in Arabidopsis. Nature Communications 9, 639. 10.1038/s41467-018-03065-7 PMC5809604 29434233

[eraf261-B75] Hepworth SR, Valverde F, Ravenscroft D, Mouradov A, Coupland G. 2002. Antagonistic regulation of flowering-time gene *SOC1* by CONSTANS and FLC via separate promoter motifs. The EMBO Journal 21, 4327–4337. 10.1093/emboj/cdf432 PMC126170 12169635

[eraf261-B76] Hisamatsu T, King RW. 2008. The nature of floral signals in Arabidopsis. II. Roles for FLOWERING LOCUS T (FT) and gibberellin. Journal of Experimental Botany 59, 3821–3829. 18931352 10.1093/jxb/ern232 PMC2576629

[eraf261-B77] Huang X, Ding J, Effgen S, Turck F, Koornneef M. 2013. Multiple loci and genetic interactions involving flowering time genes regulate stem branching among natural variants of Arabidopsis. New Phytologist 199, 843–857. 10.1111/nph.12306 23668187

[eraf261-B78] Hyun Y, Vincent C, Tilmes V, Bergonzi S, Kiefer C, Richter R, Martinez-Gallegos R, Severing E, Coupland G. 2019. A regulatory circuit conferring varied flowering response to cold in annual and perennial plants. Science 363, 409–412. 30679374 10.1126/science.aau8197

[eraf261-B79] Ibañez C, Delker C, Martinez C, et al 2018. Brassinosteroids dominate hormonal regulation of plant thermomorphogenesis via BZR1. Current Biology 28, 303–310.e3. 29337075 10.1016/j.cub.2017.11.077

[eraf261-B80] Immink RGH, Posé D, Ferrario S, et al 2012. Characterization of SOC1’s central role in flowering by the identification of its upstream and downstream regulators. Plant Physiology 160, 433–449. 10.1104/pp.112.202614 PMC3440217 22791302

[eraf261-B81] Jaeger KE, Wigge PA. 2007. FT protein acts as a long-range signal in Arabidopsis. Current Biology 17, 1050–1054. 17540569 10.1016/j.cub.2007.05.008

[eraf261-B82] Jang S, Torti S, Coupland G. 2009. Genetic and spatial interactions between FT, TSF and SVP during the early stages of floral induction in Arabidopsis. The Plant Journal 60, 614–625. 19656342 10.1111/j.1365-313X.2009.03986.x

[eraf261-B83] Jin S, Kim SY, Susila H, Nasim Z, Youn G, Ahn JH. 2022. FLOWERING LOCUS M isoforms differentially affect the subcellular localization and stability of SHORT VEGETATIVE PHASE to regulate temperature-responsive flowering in *Arabidopsis*. Molecular Plant 15, 1696–1709. 36016495 10.1016/j.molp.2022.08.007

[eraf261-B84] Johanson U, West J, Lister C, Michaels S, Amasino R, Dean C. 2000. Molecular analysis of FRIGIDA, a major determinant of natural variation in Arabidopsis flowering time. Science 290, 344–347. 10.1126/science.290.5490.344 11030654

[eraf261-B85] Jong Md, Tavares H, Pasam RK, Butler R, Ward S, George G, Melnyk CW, Challis R, Kover PX, Leyser O. 2019. Natural variation in Arabidopsis shoot branching plasticity in response to nitrate supply affects fitness. PLOS Genetics 15, e1008366. 31539368 10.1371/journal.pgen.1008366 PMC6774567

[eraf261-B86] Jung J-H, Barbosa AD, Hutin S, et al 2020. A prion-like domain in ELF3 functions as a thermosensor in Arabidopsis. Nature 585, 256–260. 10.1038/s41586-020-2644-7 32848244

[eraf261-B87] Jung J-H, Seo PJ, Ahn JH, Park C-M. 2012. Arabidopsis RNA-binding protein FCA regulates microRNA172 processing in thermosensory flowering. The Journal of Biological Chemistry 287, 16007–16016. 10.1074/jbc.M111.337485 PMC3346135 22431732

[eraf261-B89] Kim JJ, Lee JH, Kim W, Jung HS, Huijser P, Ahn JH. 2012. The microRNA156-SQUAMOSA PROMOTER BINDING PROTEIN-LIKE3 module regulates ambient temperature-responsive flowering via FLOWERING LOCUS T in Arabidopsis. Plant Physiology 159, 461–478. 22427344 10.1104/pp.111.192369 PMC3375978

[eraf261-B88] Kim W, Kim HE, Jun AR, Jung MG, Jin S, Lee JH, Ahn JH. 2016. Structural determinants of miR156a precursor processing in temperature-responsive flowering in Arabidopsis. Journal of Experimental Botany 67, 4659–4670. 27335452 10.1093/jxb/erw248 PMC4973740

[eraf261-B90] King GJ . 2015. Crop epigenetics and the molecular hardware of genotype × environment interactions. Frontiers in Plant Science 6, 968. 26594221 10.3389/fpls.2015.00968 PMC4635209

[eraf261-B91] King KE, Moritz T, Harberd NP. 2001. Gibberellins are not required for normal stem growth in *Arabidopsis thaliana* in the absence of GAI and RGA. Genetics 159, 767–776. 11606551 10.1093/genetics/159.2.767 PMC1461813

[eraf261-B92] Kinoshita A, Vayssières A, Richter R, Sang Q, Roggen A, van Driel AD, Smith RS, Coupland G. 2020. Regulation of shoot meristem shape by photoperiodic signaling and phytohormones during floral induction of Arabidopsis. eLife 9, e60661. 10.7554/eLife.60661 PMC7771970 33315012

[eraf261-B93] Kobayashi Y, Kaya H, Goto K, Iwabuchi M, Araki T. 1999. A pair of related genes with antagonistic roles in mediating flowering signals. Science 286, 1960–1962. 10583960 10.1126/science.286.5446.1960

[eraf261-B94] Komoto H, Nagahama A, Miyawaki-Kuwakado A, Hata Y, Kyozuka J, Kajita Y, Toyama H, Satake A. 2024. The transcriptional changes underlying the flowering phenology shift of *Arabidopsis halleri* in response to climate warming. Plant, Cell & Environment 47, 174–186. 10.1111/pce.14716 37691326

[eraf261-B95] Kumar SV, Lucyshyn D, Jaeger KE, Alós E, Alvey E, Harberd NP, Wigge PA. 2012. Transcription factor PIF4 controls the thermosensory activation of flowering. Nature 484, 242–245. 10.1038/nature10928 PMC4972390 22437497

[eraf261-B96] Kumar SV, Wigge PA. 2010. H2a.Z-containing nucleosomes mediate the thermosensory response in Arabidopsis. Cell 140, 136–147. 20079334 10.1016/j.cell.2009.11.006

[eraf261-B97] Kyung J, Jeon M, Jeong G, Shin Y, Seo E, Yu J, Kim H, Park C-M, Hwang D, Lee I. 2022. The two clock proteins CCA1 and LHY activate VIN3 transcription during vernalization through the vernalization-responsive cis-element. The Plant Cell 34, 1020–1037. 34931682 10.1093/plcell/koab304 PMC8894950

[eraf261-B98] Laux T, Mayer KF, Berger J, Jürgens G. 1996. The WUSCHEL gene is required for shoot and floral meristem integrity in Arabidopsis. Development 122, 87–96. 8565856 10.1242/dev.122.1.87

[eraf261-B99] Lazaro A, Obeng-Hinneh E, Albani MC. 2018. Extended vernalization regulates inflorescence fate in *Arabis alpina* by stably silencing *PERPETUAL FLOWERING1*. Plant Physiology 176, 2819–2833. 10.1104/pp.17.01754 PMC5884582 29467177

[eraf261-B103] Lee H, Yoo SJ, Lee JH, Kim W, Yoo SK, Fitzgerald H, Carrington JC, Ahn JH. 2010. Genetic framework for flowering-time regulation by ambient temperature-responsive miRNAs in Arabidopsis. Nucleic Acids Research 38, 3081–3093. 20110261 10.1093/nar/gkp1240 PMC2875011

[eraf261-B104] Lee JH, Yoo SJ, Park SH, Hwang I, Lee JS, Ahn JH. 2007. Role of SVP in the control of flowering time by ambient temperature in Arabidopsis. Genes & Development 21, 397–402. 17322399 10.1101/gad.1518407 PMC1804328

[eraf261-B102] Lee JH, Ryu H-S, Chung KS, Posé D, Kim S, Schmid M, Ahn JH. 2013. Regulation of temperature-responsive flowering by MADS-box transcription factor repressors. Science 342, 628–632. 10.1126/science.1241097 24030492

[eraf261-B101] Lee KP, Piskurewicz U, Turečková V, Strnad M, Lopez-Molina L. 2010. A seed coat bedding assay shows that RGL2-dependent release of abscisic acid by the endosperm controls embryo growth in Arabidopsis dormant seeds. Proceedings of the National Academy of Sciences, USA 107, 19108–19113. 10.1073/pnas.1012896107 PMC2973907 20956298

[eraf261-B100] Lee S, Cheng H, King KE, Wang W, He Y, Hussain A, Lo J, Harberd NP, Peng J. 2002. Gibberellin regulates Arabidopsis seed germination via RGL2, a GAI/RGA-like gene whose expression is up-regulated following imbibition. Genes & Development 16, 646–658. 11877383 10.1101/gad.969002 PMC155355

[eraf261-B105] Leida C, Conesa A, Llácer G, Badenes ML, Ríos G. 2012. Histone modifications and expression of *DAM6* gene in peach are modulated during bud dormancy release in a cultivar-dependent manner. New Phytologist 193, 67–80. 10.1111/j.1469-8137.2011.03863.x 21899556

[eraf261-B106] Lempe J, Balasubramanian S, Sureshkumar S, Singh A, Schmid M, Weigel D. 2005. Diversity of flowering responses in wild Arabidopsis thaliana strains. PLOS Genetics 1, 109–118. 16103920 10.1371/journal.pgen.0010006 PMC1183525

[eraf261-B107] Li D, Liu C, Shen L, Wu Y, Chen H, Robertson M, Helliwell CA, Ito T, Meyerowitz E, Yu H. 2008. A repressor complex governs the integration of flowering signals in *Arabidopsis*. Developmental Cell 15, 110–120. 18606145 10.1016/j.devcel.2008.05.002

[eraf261-B108] Liu C, Chen H, Er HL, Soo HM, Kumar PP, Han J-H, Liou YC, Yu H. 2008. Direct interaction of AGL24 and SOC1 integrates flowering signals in Arabidopsis. Development 135, 1481–1491. 18339670 10.1242/dev.020255

[eraf261-B110] Liu C, Zhou J, Bracha-Drori K, Yalovsky S, Ito T, Yu H. 2007. Specification of *Arabidopsis* floral meristem identity by repression of flowering time genes. Development 134, 1901–1910. 10.1242/dev.003103 17428825

[eraf261-B109] Liu L, Zhang Y, Yu H. 2020. Florigen trafficking integrates photoperiod and temperature signals in Arabidopsis. Journal of Integrative Plant Biology 62, 1385–1398. 32729982 10.1111/jipb.13000

[eraf261-B111] Lomas J, Burd P. 1983. Prediction of the commencement and duration of the flowering period of citrus. Agricultural Meteorology 28, 387–396.

[eraf261-B112] Lu X, O’Neill CM, Warner S, Xiong Q, Chen X, Wells R, Penfield S. 2022. Winter warming post floral initiation delays flowering via bud dormancy activation and affects yield in a winter annual crop. Proceedings of the National Academy of Sciences, USA 119, e2204355119. 10.1073/pnas.2204355119 PMC9522361 36122201

[eraf261-B113] Lu Z, Yu H, Xiong G, et al 2013. Genome-wide binding analysis of the transcription activator IDEAL PLANT ARCHITECTURE1 reveals a complex network regulating rice plant architecture. The Plant Cell 25, 3743–3759. 24170127 10.1105/tpc.113.113639 PMC3877814

[eraf261-B114] Mandel MA, Yanofsky MF. 1995. A gene triggering flower formation in Arabidopsis. Nature 377, 522–524. 10.1038/377522a0 7566148

[eraf261-B115] Marín-González E, Matías-Hernández L, Aguilar-Jaramillo AE, Lee JH, Ahn JH, Suárez-López P, Pelaz S. 2015. SHORT VEGETATIVE PHASE up-regulates TEMPRANILLO2 floral repressor at low ambient temperatures. Plant Physiology 169, 1214–1224. 10.1104/pp.15.00570 PMC4587448 26243615

[eraf261-B116] Martín-Fontecha ES, Tarancón C, Cubas P. 2018. To grow or not to grow, a power-saving program induced in dormant buds. Current Opinion in Plant Biology 41, 102–109. 29125947 10.1016/j.pbi.2017.10.001

[eraf261-B117] Matar S, Kumar A, Holtgräwe D, Weisshaar B, Melzer S. 2021. The transition to flowering in winter rapeseed during vernalization. Plant, Cell & Environment 44, 506–518. 10.1111/pce.13946 33190312

[eraf261-B118] Mateos JL, Madrigal P, Tsuda K, Rawat V, Richter R, Romera-Branchat M, Fornara F, Schneeberger K, Krajewski P, Coupland G. 2015. Combinatorial activities of SHORT VEGETATIVE PHASE and FLOWERING LOCUS C define distinct modes of flowering regulation in Arabidopsis. Genome Biology 16, 31. 25853185 10.1186/s13059-015-0597-1 PMC4378019

[eraf261-B119] Mateos JL, Tilmes V, Madrigal P, Severing E, Richter R, Rijkenberg CWM, Krajewski P, Coupland G. 2017. Divergence of regulatory networks governed by the orthologous transcription factors FLC and PEP1 in Brassicaceae species. Proceedings of the National Academy of Sciences, USA 114, E11037–E11046. 10.1073/pnas.1618075114 PMC5754749 29203652

[eraf261-B120] Mathieu J, Warthmann N, Küttner F, Schmid M. 2007. Export of FT protein from phloem companion cells is sufficient for floral induction in *Arabidopsis*. Current Biology 17, 1055–1060. 17540570 10.1016/j.cub.2007.05.009

[eraf261-B121] Mayer KF, Schoof H, Haecker A, Lenhard M, Jürgens G, Laux T. 1998. Role of WUSCHEL in regulating stem cell fate in the Arabidopsis shoot meristem. Cell 95, 805–815. 10.1016/s0092-8674(00)81703-1 9865698

[eraf261-B122] McKim SM . 2020. Moving on up—controlling internode growth. New Phytologist 226, 672–678. 10.1111/nph.16439 31955426

[eraf261-B123] Melzer S, Lens F, Gennen J, Vanneste S, Rohde A, Beeckman T. 2008. Flowering-time genes modulate meristem determinacy and growth form in *Arabidopsis thaliana*. Nature Genetics 40, 1489–1492. 18997783 10.1038/ng.253

[eraf261-B124] Merelo P, González-Cuadra I, Ferrándiz C. 2022. A cellular analysis of meristem activity at the end of flowering points to cytokinin as a major regulator of proliferative arrest in Arabidopsis. Current Biology 32, 749–762.e3. 10.1016/j.cub.2021.11.069 34963064

[eraf261-B125] Mesejo C, Marzal A, Martínez-Fuentes A, Reig C, de Lucas M, Iglesias DJ, Primo-Millo E, Blázquez MA, Agustí M. 2022. Reversion of fruit-dependent inhibition of flowering in Citrus requires sprouting of buds with epigenetically silenced *CcMADS19*. New Phytologist 233, 526–533. 10.1111/nph.17681 34403516

[eraf261-B126] Michaels SD, Amasino RM. 1999. *FLOWERING LOCUS C* encodes a novel MADS domain protein that acts as a repressor of flowering. The Plant Cell 11, 949–956. 10330478 10.1105/tpc.11.5.949 PMC144226

[eraf261-B127] Min Y, Kramer EM. 2023. All’s well that ends well: the timing of floral meristem termination. New Phytologist 238, 500–505. 36600362 10.1111/nph.18715

[eraf261-B128] Miryeganeh M . 2020. Synchronization of senescence and desynchronization of flowering in Arabidopsis thaliana. AoB PLANTS 12, plaa018. 10.1093/aobpla/plaa018 PMC7299267 32577195

[eraf261-B129] Moon J, Suh S-S, Lee H, Choi K-R, Hong CB, Paek N-C, Kim S-G, Lee I. 2003. The SOC1 MADS-box gene integrates vernalization and gibberellin signals for flowering in Arabidopsis. The Plant Journal 35, 613–623. 12940954 10.1046/j.1365-313x.2003.01833.x

[eraf261-B130] Murase K, Hirano Y, Sun T, Hakoshima T. 2008. Gibberellin-induced DELLA recognition by the gibberellin receptor GID1. Nature 456, 459–463. 10.1038/nature07519 19037309

[eraf261-B131] Mutasa-Göttgens E, Hedden P. 2009. Gibberellin as a factor in floral regulatory networks. Journal of Experimental Botany 60, 1979–1989. 10.1093/jxb/erp040 19264752

[eraf261-B132] Nagahama A, Kubota Y, Satake A. 2018. Climate warming shortens flowering duration: a comprehensive assessment of plant phenological responses based on gene expression analyses and mathematical modeling. Ecological Research 33, 1059–1068.

[eraf261-B133] Nagano AJ, Kawagoe T, Sugisaka J, Honjo MN, Iwayama K, Kudoh H. 2019. Annual transcriptome dynamics in natural environments reveals plant seasonal adaptation. Nature Plants 5, 74–83. 30617252 10.1038/s41477-018-0338-z

[eraf261-B134] Niwa M, Daimon Y, Kurotani K, et al 2013. BRANCHED1 interacts with FLOWERING LOCUS T to repress the floral transition of the axillary meristems in Arabidopsis. The Plant Cell 25, 1228–1242. 23613197 10.1105/tpc.112.109090 PMC3663264

[eraf261-B135] Nusinow DA, Helfer A, Hamilton EE, King JJ, Imaizumi T, Schultz TF, Farré EM, Kay SA. 2011. The ELF4–ELF3–LUX complex links the circadian clock to diurnal control of hypocotyl growth. Nature 475, 398–402. 10.1038/nature10182 PMC3155984 21753751

[eraf261-B136] O’Neill CM, Lu X, Calderwood A, Tudor EH, Robinson P, Wells R, Morris R, Penfield S. 2019. Vernalization and floral transition in autumn drive winter annual life history in oilseed rape. Current Biology 29, 4300–4306.e2. 31813609 10.1016/j.cub.2019.10.051 PMC6926474

[eraf261-B137] Ongaro V, Bainbridge K, Williamson L, Leyser O. 2008. Interactions between axillary branches of Arabidopsis. Molecular Plant 1, 388–400. 10.1093/mp/ssn007 19825548

[eraf261-B138] Osnato M, Castillejo C, Matías-Hernández L, Pelaz S. 2012. *TEMPRANILLO* genes link photoperiod and gibberellin pathways to control flowering in Arabidopsis. Nature Communications 3, 808. 10.1038/ncomms1810 22549837

[eraf261-B139] Pajoro A, Biewers S, Dougali E, et al 2014. The (r)evolution of gene regulatory networks controlling Arabidopsis plant reproduction: a two-decade history. Journal of Experimental Botany 65, 4731–4745. 10.1093/jxb/eru233 24913630

[eraf261-B140] Penfield S . 2024. Beyond floral initiation: the role of flower bud dormancy in flowering time control of annual plants. Journal of Experimental Botany 75, 6056–6062. 38795335 10.1093/jxb/erae223 PMC11480682

[eraf261-B141] Peng J, Carol P, Richards DE, King KE, Cowling RJ, Murphy GP, Harberd NP. 1997. The Arabidopsis *GAI* gene defines a signaling pathway that negatively regulates gibberellin responses. Genes & Development 11, 3194–3205. 9389651 10.1101/gad.11.23.3194 PMC316750

[eraf261-B142] Périlleux C, Bouché F, Randoux M, Orman-Ligeza B. 2019. Turning meristems into fortresses. Trends in Plant Science 24, 431–442. 10.1016/j.tplants.2019.02.004 30853243

[eraf261-B143] Porri A, Torti S, Romera-Branchat M, Coupland G. 2012. Spatially distinct regulatory roles for gibberellins in the promotion of flowering of Arabidopsis under long photoperiods. Development 139, 2198–2209. 10.1242/dev.077164 22573618

[eraf261-B144] Posé D, Verhage L, Ott F, Yant L, Mathieu J, Angenent GC, Immink RGH, Schmid M. 2013. Temperature-dependent regulation of flowering by antagonistic FLM variants. Nature 503, 414–417. 24067612 10.1038/nature12633

[eraf261-B145] Prusinkiewicz P, Crawford S, Smith RS, Ljung K, Bennett T, Ongaro V, Leyser O. 2009. Control of bud activation by an auxin transport switch. Proceedings of the National Academy of Sciences, USA 106, 17431–17436. 10.1073/pnas.0906696106 PMC2751654 19805140

[eraf261-B146] Prusinkiewicz P, Erasmus Y, Lane B, Harder LD, Coen E. 2007. Evolution and development of inflorescence architectures. Science 316, 1452–1456. 10.1126/science.1140429 17525303

[eraf261-B147] Quesada-Traver C, Lloret A, Carretero-Paulet L, Badenes ML, Ríos G. 2022. Evolutionary origin and functional specialization of Dormancy-Associated MADS box (DAM) proteins in perennial crops. BMC Plant Biology 22, 473. 10.1186/s12870-022-03856-7 PMC9533583 36199018

[eraf261-B148] Ratcliffe OJ, Amaya I, Vincent CA, Rothstein S, Carpenter R, Coen ES, Bradley DJ. 1998. A common mechanism controls the life cycle and architecture of plants. Development 125, 1609–1615. 9521899 10.1242/dev.125.9.1609

[eraf261-B149] Rieu I, Ruiz-Rivero O, Fernandez-Garcia N, et al 2008. The gibberellin biosynthetic genes *AtGA20ox1* and *AtGA20ox2* act, partially redundantly, to promote growth and development throughout the Arabidopsis life cycle. The Plant Journal 53, 488–504. 10.1111/j.1365-313X.2007.03356.x 18069939

[eraf261-B150] Rinne PLH, Welling A, Vahala J, Ripel L, Ruonala R, Kangasjärvi J, van der Schoot C. 2011. Chilling of dormant buds hyperinduces FLOWERING LOCUS T and recruits GA-inducible 1,3-β-glucanases to reopen signal conduits and release dormancy in Populus. The Plant Cell 23, 130–146. 10.1105/tpc.110.081307 PMC3051240 21282527

[eraf261-B151] Sadik S . 1962. Morphology of the curd of cauliflower. American Journal of Botany 49, 290–297.

[eraf261-B152] Salam BB, Barbier F, Danieli R, et al 2021. Sucrose promotes stem branching through cytokinin. Plant Physiology 185, 1708–1721. 33793932 10.1093/plphys/kiab003 PMC8133652

[eraf261-B153] Samach A, Onouchi H, Gold SE, Ditta GS, Schwarz-Sommer Z, Yanofsky MF, Coupland G. 2000. Distinct roles of CONSTANS target genes in reproductive development of *Arabidopsis*. Science 288, 1613–1616. 10834834 10.1126/science.288.5471.1613

[eraf261-B154] Sánchez-Gerschon V, Martínez-Fernández I, González-Bermúdez MR, de la Hoz-Rodríguez S, González FV, Lozano-Juste J, Ferrándiz C, Balanzà V. 2024. Transcription factors HB21/40/53 trigger inflorescence arrest through abscisic acid accumulation at the end of flowering. Plant Physiology 195, 2743–2756. 38669447 10.1093/plphys/kiae234 PMC11288733

[eraf261-B155] Satake A, Kawagoe T, Saburi Y, Chiba Y, Sakurai G, Kudoh H. 2013. Forecasting flowering phenology under climate warming by modelling the regulatory dynamics of flowering-time genes. Nature Communications 4, 2303. 10.1038/ncomms3303 23941973

[eraf261-B156] Schmid M, Uhlenhaut NH, Godard F, Demar M, Bressan R, Weigel D, Lohmann JU. 2003. Dissection of floral induction pathways using global expression analysis. Development 130, 6001–6012. 14573523 10.1242/dev.00842

[eraf261-B157] Schoof H, Lenhard M, Haecker A, Mayer KFX, Jürgens G, Laux T. 2000. The stem cell population of *Arabidopsis* shoot meristems is maintained by a regulatory loop between the *CLAVATA* and *WUSCHEL* genes. Cell 100, 635–644. 10761929 10.1016/s0092-8674(00)80700-x

[eraf261-B158] Schultz EA, Haughn GW. 1991. LEAFY, a homeotic gene that regulates inflorescence development in Arabidopsis. The Plant Cell 3, 771–781. 12324613 10.1105/tpc.3.8.771 PMC160044

[eraf261-B159] Searle I, He Y, Turck F, Vincent C, Fornara F, Kröber S, Amasino RA, Coupland G. 2006. The transcription factor FLC confers a flowering response to vernalization by repressing meristem competence and systemic signaling in Arabidopsis. Genes & Development 20, 898–912. 10.1101/gad.373506 PMC1472290 16600915

[eraf261-B160] Serrano-Mislata A, Goslin K, Zheng B, Rae L, Wellmer F, Graciet E, Madueño F. 2017. Regulatory interplay between LEAFY, APETALA1/CAULIFLOWER and TERMINAL FLOWER1: new insights into an old relationship. Plant Signaling & Behavior 12, e1370164. 28873010 10.1080/15592324.2017.1370164 PMC5647955

[eraf261-B161] Shen Y, Lei T, Cui X, Liu X, Zhou S, Zheng Y, Guérard F, Issakidis-Bourguet E, Zhou D-X. 2019. Arabidopsis histone deacetylase HDA15 directly represses plant response to elevated ambient temperature. The Plant Journal 100, 991–1006. 10.1111/tpj.14492 31400169

[eraf261-B162] Shikata M, Koyama T, Mitsuda N, Ohme-Takagi M. 2009. Arabidopsis SBP-box genes SPL10, SPL11 and SPL2 control morphological change in association with shoot maturation in the reproductive phase. Plant & Cell Physiology 50, 2133–2145. 19880401 10.1093/pcp/pcp148

[eraf261-B163] Shimada A, Ueguchi-Tanaka M, Nakatsu T, Nakajima M, Naoe Y, Ohmiya H, Kato H, Matsuoka M. 2008. Structural basis for gibberellin recognition by its receptor GID1. Nature 456, 520–523. 10.1038/nature07546 19037316

[eraf261-B164] Shindo C, Aranzana MJ, Lister C, Baxter C, Nicholls C, Nordborg M, Dean C. 2005. Role of FRIGIDA and FLOWERING LOCUS C in determining variation in flowering time of Arabidopsis. Plant Physiology 138, 1163–1173. 15908596 10.1104/pp.105.061309 PMC1150429

[eraf261-B165] Silverstone AL, Jung H-S, Dill A, Kawaide H, Kamiya Y, Sun T. 2001. Repressing a repressor: gibberellin-induced rapid reduction of the RGA protein in Arabidopsis. The Plant Cell 13, 1555–1566. 10.1105/TPC.010047 PMC139546 11449051

[eraf261-B166] Singh RK, Maurya JP, Azeez A, Miskolczi P, Tylewicz S, Stojkovič K, Delhomme N, Busov V, Bhalerao RP. 2018. A genetic network mediating the control of bud break in hybrid aspen. Nature Communications 9, 4173. 10.1038/s41467-018-06696-y PMC6177393 30301891

[eraf261-B167] Singh RK, Miskolczi P, Maurya JP, Bhalerao RP. 2019. A tree ortholog of SHORT VEGETATIVE PHASE floral repressor mediates photoperiodic control of bud dormancy. Current Biology: CB 29, 128–133.e2. 30554900 10.1016/j.cub.2018.11.006

[eraf261-B168] Song X, Lu Z, Yu H, et al 2017. IPA1 functions as a downstream transcription factor repressed by D53 in strigolactone signaling in rice. Cell Research 27, 1128–1141. 28809396 10.1038/cr.2017.102 PMC5587847

[eraf261-B169] Song YH, Shim JS, Kinmonth-Schultz HA, Imaizumi T. 2015. Photoperiodic flowering: time measurement mechanisms in leaves. Annual Review of Plant Biology 66, 441–464. 10.1146/annurev-arplant-043014-115555 PMC4414745 25534513

[eraf261-B170] Sriboon S, Li H, Guo C, Senkhamwong T, Dai C, Liu K. 2020. Knock-out of *TERMINAL FLOWER 1* genes altered flowering time and plant architecture in *Brassica napus*. BMC Genetics 21, 52. 32429836 10.1186/s12863-020-00857-z PMC7236879

[eraf261-B171] Srikanth A, Schmid M. 2011. Regulation of flowering time: all roads lead to Rome. Cellular and Molecular Life Sciences 68, 2013–2037. 21611891 10.1007/s00018-011-0673-y PMC11115107

[eraf261-B172] Suh S-S, Choi K-R, Lee I. 2003. Revisiting phase transition during flowering in Arabidopsis. Plant & Cell Physiology 44, 836–843. 12941876 10.1093/pcp/pcg109

[eraf261-B173] Sung S, Amasino RM. 2004. Vernalization in Arabidopsis thaliana is mediated by the PHD finger protein VIN3. Nature 427, 159–164. 10.1038/nature02195 14712276

[eraf261-B174] Sureshkumar S, Dent C, Seleznev A, Tasset C, Balasubramanian S. 2016. Nonsense-mediated mRNA decay modulates FLM-dependent thermosensory flowering response in Arabidopsis. Nature Plants 2, 1–7. 10.1038/nplants.2016.55 27243649

[eraf261-B175] Susila H, Gawarecka K, Youn G, Jurić S, Jeong H, Ahn JH. 2024. THYLAKOID FORMATION 1 interacts with FLOWERING LOCUS T and modulates temperature-responsive flowering in Arabidopsis. The Plant Journal 120, 60–75. 10.1111/tpj.16970 39136360

[eraf261-B176] Susila H, Jurić S, Liu L, et al 2021. Florigen sequestration in cellular membranes modulates temperature-responsive flowering. Science 373, 1137–1142. 10.1126/science.abh4054 34516842

[eraf261-B177] Susila H, Nasim Z, Ahn J. 2018. Ambient temperature-responsive mechanisms coordinate regulation of flowering time. International Journal of Molecular Sciences 19, 3196. 10.3390/ijms19103196 PMC6214042 30332820

[eraf261-B178] Tanaka M, Takei K, Kojima M, Sakakibara H, Mori H. 2006. Auxin controls local cytokinin biosynthesis in the nodal stem in apical dominance. The Plant Journal 45, 1028–1036. 16507092 10.1111/j.1365-313X.2006.02656.x

[eraf261-B179] Tao Z, Shen L, Liu C, Liu L, Yan Y, Yu H. 2012. Genome-wide identification of SOC1 and SVP targets during the floral transition in Arabidopsis. The Plant Journal 70, 549–561. 22268548 10.1111/j.1365-313X.2012.04919.x

[eraf261-B180] Tasset C, Yadav AS, Sureshkumar S, Singh R, Woude Lvd, Nekrasov M, Tremethick D, Zanten Mv, Balasubramanian S. 2018. POWERDRESS-mediated histone deacetylation is essential for thermomorphogenesis in *Arabidopsis thaliana*. PLOS Genetics 14, e1007280. 10.1371/journal.pgen.1007280 PMC5874081 29547672

[eraf261-B181] Teper-Bamnolker P, Samach A. 2005. The flowering integrator FT regulates SEPALLATA3 and FRUITFULL accumulation in Arabidopsis leaves. The Plant Cell 17, 2661–2675. 10.1105/tpc.105.035766 PMC1242264 16155177

[eraf261-B182] Thines BC, Youn Y, Duarte MI, Harmon FG. 2014. The time of day effects of warm temperature on flowering time involve PIF4 and PIF5. Journal of Experimental Botany 65, 1141–1151. 24574484 10.1093/jxb/ert487 PMC3935576

[eraf261-B183] Torti S, Fornara F, Vincent C, Andrés F, Nordström K, Göbel U, Knoll D, Schoof H, Coupland G. 2012. Analysis of the Arabidopsis shoot meristem transcriptome during floral transition identifies distinct regulatory patterns and a leucine-rich repeat protein that promotes flowering. The Plant Cell 24, 444–462. 10.1105/tpc.111.092791 PMC3315226 22319055

[eraf261-B184] Tyler L, Thomas SG, Hu J, Dill A, Alonso JM, Ecker JR, Sun T. 2004. DELLA proteins and gibberellin-regulated seed germination and floral development in Arabidopsis. Plant Physiology 135, 1008–1019. 10.1104/pp.104.039578 PMC514135 15173565

[eraf261-B185] Tylewicz S, Petterle A, Marttila S, et al 2018. Photoperiodic control of seasonal growth is mediated by ABA acting on cell-cell communication. Science 360, 212–215. 10.1126/science.aan8576 29519919

[eraf261-B186] Ueguchi-Tanaka M, Ashikari M, Nakajima M, et al 2005. *GIBBERELLIN INSENSITIVE DWARF1* encodes a soluble receptor for gibberellin. Nature 437, 693–698. 16193045 10.1038/nature04028

[eraf261-B187] van der Woude LC, Perrella G, Snoek BL, et al 2019. HISTONE DEACETYLASE 9 stimulates auxin-dependent thermomorphogenesis in Arabidopsis thaliana by mediating H2A.Z depletion. Proceedings of the National Academy of Sciences, USA 116, 25343–25354. 10.1073/pnas.1911694116 PMC6911240 31767749

[eraf261-B188] Vayssières A, Mishra P, Roggen A, Neumann U, Ljung K, Albani MC. 2020. Vernalization shapes shoot architecture and ensures the maintenance of dormant buds in the perennial *Arabis alpina*. New Phytologist 227, 99–115. 32022273 10.1111/nph.16470

[eraf261-B189] Wahl V, Brand LH, Guo Y-L, Schmid M. 2010. The FANTASTIC FOUR proteins influence shoot meristem size in *Arabidopsis thaliana*. BMC Plant Biology 10, 285. 21176196 10.1186/1471-2229-10-285 PMC3023791

[eraf261-B190] Wahl V, Ponnu J, Schlereth A, Arrivault S, Langenecker T, Franke A, Feil R, Lunn JE, Stitt M, Schmid M. 2013. Regulation of flowering by trehalose-6-phosphate signaling in Arabidopsis thaliana. Science 339, 704–707. 23393265 10.1126/science.1230406

[eraf261-B191] Walker CH, Ware A, Šimura J, Ljung K, Wilson Z, Bennett T. 2023. Cytokinin signaling regulates two-stage inflorescence arrest in Arabidopsis. Plant Physiology 191, 479–495. 10.1093/plphys/kiac514 PMC9806609 36331332

[eraf261-B193] Wang J-W, Czech B, Weigel D. 2009. miR156-regulated SPL transcription factors define an endogenous flowering pathway in Arabidopsis thaliana. Cell 138, 738–749. 19703399 10.1016/j.cell.2009.06.014

[eraf261-B192] Wang R, Albani MC, Vincent C, Bergonzi S, Luan M, Bai Y, Kiefer C, Castillo R, Coupland G. 2011. *AaTFL1* confers an age-dependent response to vernalization in perennial *Arabis alpina*. The Plant Cell 23, 1307–1321. 21498681 10.1105/tpc.111.083451 PMC3101554

[eraf261-B194] Wang R, Farrona S, Vincent C, Joecker A, Schoof H, Turck F, Alonso-Blanco C, Coupland G, Albani MC. 2009. PEP1 regulates perennial flowering in *Arabis alpina*. Nature 459, 423–427. 10.1038/nature07988 19369938

[eraf261-B195] Wang L, Wang B, Jiang L, Liu X, Li X, Lu Z, Meng X, Wang Y, Smith SM, Li J. 2015. Strigolactone signaling in Arabidopsis regulates shoot development by targeting D53-like SMXL repressor proteins for ubiquitination and degradation. The Plant Cell 27, 3128–3142. 26546446 10.1105/tpc.15.00605 PMC4682305

[eraf261-B196] Ware A, Walker CH, Šimura J, González-Suárez P, Ljung K, Bishopp A, Wilson ZA, Bennett T. 2020. Auxin export from proximal fruits drives arrest in temporally competent inflorescences. Nature Plants 6, 699–707. 10.1038/s41477-020-0661-z 32451444

[eraf261-B197] Whittaker C, Dean C. 2017. The FLC locus: a platform for discoveries in epigenetics and adaptation. Annual Review of Cell and Developmental Biology 33, 555–575. 10.1146/annurev-cellbio-100616-060546 28693387

[eraf261-B198] Wigge PA, Kim MC, Jaeger KE, Busch W, Schmid M, Lohmann JU, Weigel D. 2005. Integration of spatial and temporal information during floral induction in Arabidopsis. Science 309, 1056–1059. 10.1126/science.1114358 16099980

[eraf261-B199] Willige BC, Ghosh S, Nill C, Zourelidou M, Dohmann EMN, Maier A, Schwechheimer C. 2007. The DELLA domain of GA INSENSITIVE mediates the interaction with the GA INSENSITIVE DWARF1A gibberellin receptor of Arabidopsis. The Plant Cell 19, 1209–1220. 10.1105/tpc.107.051441 PMC1913748 17416730

[eraf261-B200] Wilson RN, Heckman JW, Somerville CR. 1992. Gibberellin is Required for Flowering in Arabidopsis thaliana under Short Days. Plant Physiology 100, 403–408. 10.1104/pp.100.1.403 PMC1075565 16652976

[eraf261-B201] Wollenberg AC, Amasino RM. 2012. Natural variation in the temperature range permissive for vernalization in accessions of Arabidopsis thaliana. Plant, Cell & Environment 35, 2181–2191. 10.1111/j.1365-3040.2012.02548.x 22639792

[eraf261-B202] Wu G, Park MY, Conway SR, Wang J-W, Weigel D, Poethig RS. 2009. The sequential action of miR156 and miR172 regulates developmental timing in Arabidopsis. Cell 138, 750–759. 10.1016/j.cell.2009.06.031 PMC2732587 19703400

[eraf261-B203] Wu G, Poethig RS. 2006. Temporal regulation of shoot development in Arabidopsis thaliana by miR156 and its target SPL3. Development 133, 3539–3547. 10.1242/dev.02521 PMC1610107 16914499

[eraf261-B204] Wu R, Tomes S, Karunairetnam S, Tustin SD, Hellens RP, Allan AC, Macknight RC, Varkonyi-Gasic E. 2017. SVP-like MADS box genes control dormancy and budbreak in apple. Frontiers in Plant Science 8, 477. 10.3389/fpls.2017.00477 PMC5378812 28421103

[eraf261-B205] Xie Y, Liu Y, Ma M, Zhou Q, Zhao Y, Zhao B, Wang B, Wei H, Wang H. 2020. Arabidopsis FHY3 and FAR1 integrate light and strigolactone signaling to regulate branching. Nature Communications 11, 1955. 10.1038/s41467-020-15893-7 PMC7181604 32327664

[eraf261-B206] Yamaguchi A, Kobayashi Y, Goto K, Abe M, Araki T. 2005. TWIN SISTER OF FT (TSF) acts as a floral pathway integrator redundantly with FT. Plant & Cell Physiology 46, 1175–1189. 10.1093/pcp/pci151 15951566

[eraf261-B208] Yamaguchi A, Wu M-F, Yang L, Wu G, Poethig RS, Wagner D. 2009. The MicroRNA-regulated SBP-box transcription factor SPL3 is a direct upstream activator of *LEAFY*, *FRUITFULL*, and *APETALA1*. Developmental Cell 17, 268–278. 10.1016/j.devcel.2009.06.007 PMC2908246 19686687

[eraf261-B207] Yamaguchi N, Winter CM, Wu M-F, Kanno Y, Yamaguchi A, Seo M, Wagner D. 2014. Gibberellin acts positively then negatively to control onset of flower formation in Arabidopsis. Science 344, 638–641. 24812402 10.1126/science.1250498

[eraf261-B209] Yoo SK, Chung KS, Kim J, Lee JH, Hong SM, Yoo SJ, Yoo SY, Lee JS, Ahn JH. 2005. CONSTANS activates SUPPRESSOR OF OVEREXPRESSION OF CONSTANS 1 through FLOWERING LOCUS T to promote flowering in Arabidopsis. Plant Physiology 139, 770–778. 16183837 10.1104/pp.105.066928 PMC1255994

[eraf261-B210] Yu S, Galvão VC, Zhang Y-C, Horrer D, Zhang T-Q, Hao Y-H, Feng Y-Q, Wang S, Schmid M, Wang J-W. 2012. Gibberellin regulates the Arabidopsis floral transition through miR156-targeted SQUAMOSA PROMOTER BINDING–LIKE transcription factors. The Plant Cell 24, 3320–3332. 22942378 10.1105/tpc.112.101014 PMC3462634

[eraf261-B211] Zhao Y, Antoniou-Kourounioti RL, Calder G, Dean C, Howard M. 2020. Temperature-dependent growth contributes to long-term cold sensing. Nature 583, 825–829. 32669706 10.1038/s41586-020-2485-4 PMC7116785

[eraf261-B212] Zhao Y, Zhu P, Hepworth J, Bloomer R, Antoniou-Kourounioti RL, Doughty J, Heckmann A, Xu C, Yang H, Dean C. 2021. Natural temperature fluctuations promote COOLAIR regulation of FLC. Genes & Development 35, 888–898. 10.1101/gad.348362.121 PMC8168555 33985972

[eraf261-B213] Zhu H, Chen P-Y, Zhong S, et al 2020. Thermal-responsive genetic and epigenetic regulation of DAM cluster controlling dormancy and chilling requirement in peach floral buds. Horticulture Research 7, 114. 32821397 10.1038/s41438-020-0336-y PMC7395172

[eraf261-B215] Zhu P, Lister C, Dean C. 2021. Cold-induced Arabidopsis FRIGIDA nuclear condensates for FLC repression. Nature 599, 657–661. 10.1038/s41586-021-04062-5 PMC8612926 34732891

[eraf261-B217] Zhu T, Zanten Mv, Smet ID. 2022. Wandering between hot and cold: temperature dose-dependent responses. Trends in Plant Science 27, 1124–1133. 35810070 10.1016/j.tplants.2022.06.001

[eraf261-B214] Zhu Y, Klasfeld S, Jeong CW, Jin R, Goto K, Yamaguchi N, Wagner D. 2020. TERMINAL FLOWER 1-FD complex target genes and competition with FLOWERING LOCUS T. Nature Communications 11, 5118. 10.1038/s41467-020-18782-1 PMC7550357 33046692

[eraf261-B216] Zhu Y, Wagner D. 2020. Plant inflorescence architecture: the formation, activity, and fate of axillary meristems. Cold Spring Harbor Perspectives in Biology 12, a034652. 31308142 10.1101/cshperspect.a034652 PMC6942122

[eraf261-B218] Zsögön A, Čermák T, Naves ER, Notini MM, Edel KH, Weinl S, Freschi L, Voytas DF, Kudla J, Peres LEP. 2018. *De novo* domestication of wild tomato using genome editing. Nature Biotechnology 36, 1211–1216. 10.1038/nbt.4272 30272678

